# Healthcare seeking for chronic illness among adult slum dwellers in Bangladesh: A descriptive cross-sectional study in two urban settings

**DOI:** 10.1371/journal.pone.0233635

**Published:** 2020-06-15

**Authors:** Alayne M. Adams, Rubana Islam, Sifat Shahana Yusuf, Anthony Panasci, Nancy Crowell

**Affiliations:** 1 Department of Family Medicine, McGill University, Montreal, Canada; 2 Health Systems and Population Studies Division, icddr,b, Dhaka, Bangladesh; 3 School of Public Health & Community Medicine, University of New South Wales, Sydney, Australia; 4 Department of Global Health, School of Nursing and Health Studies, Georgetown University, Washington, District of Columbia, United States of America; 5 School of Nursing and Health Studies, Georgetown University, Washington, District of Columbia, United States of America; Anglia Ruskin University, UNITED KINGDOM

## Abstract

**Introduction:**

Accompanying rapid urbanization in Bangladesh are inequities in health and healthcare which are most visibly manifested in slums or low-income settlements. This study examines socioeconomic, demographic and geographic patterns of self-reported chronic illness and healthcare seeking among adult slum dwellers in Bangladesh. Understanding these patterns is critical in designing more equitable urban health systems and in enabling the country’s goal of Universal Health Coverage by 2030.

**Methods:**

This descriptive cross-sectional study compares survey data from slum settlements located in two urban sites in Bangladesh, Tongi and Sylhet. Reported chronic illness symptoms and associated healthcare-seeking strategies are compared, and the catastrophic impact of household healthcare expenditures are assessed.

**Results:**

Significant differences in healthcare-seeking for chronic illness were apparent both within and between slum settlements related to sex, wealth score (PPI), and location. Women were more likely to use private clinics than men. Compared to poorer residents, those from wealthier households sought care to a greater extent in private clinics, while poorer households relied more on drug shops and public hospitals. Chronic symptoms also differed. A greater prevalence of musculoskeletal, respiratory, digestive and neurological symptoms was reported among those with lower PPIs. In both slum sites, reliance on the private healthcare market was widespread, but greater in industrialized Tongi. Tongi also experienced a higher probability of catastrophic expenditure than Sylhet.

**Conclusions:**

Study results point to the value of understanding context-specific health-seeking patterns for chronic illness when designing delivery strategies to address the growing burden of NCDs in slum environments. Slums are complex social and geographic entities and cannot be generalized. Priority attention should be focused on developing chronic care services that meet the needs of the working poor in terms of proximity, opening hours, quality, and cost.

## Introduction

Urbanization is a global phenomenon with 55% of the global population living in urban areas. Rapid population growth in urban areas of Asia and Africa will bring this figure closer to 70% by 2050 [1], the bulk of which will concentrate in slums or low-income settlements [2] characterized by crowding, insecurity, inadequate housing and limited access to basic services. [3] With urbanization comes economic development and greater availability of healthcare services. [4] However, the urban advantage is not enjoyed uniformly by all urban residents. [5] Consistent with Julian Tudor Hart’s (1971) inverse-care law [6], evidence suggests that the better-off disproportionately benefit from urban healthcare access while poorer citizens are more likely to experience unhealthy environments and poorer health outcomes. [5] The adverse physical and social conditions of slums are inimical to the SDG Goal 3 of “ensuing healthy lives for all” [3,7], as are many barriers slum dwellers face in accessing quality health services including the high costs of care, lack of proximity, limited hours of service, overburdened facilities, as well as a perceived lack of respectful and effective treatment. [8–11]

In Bangladesh, approximately 55% of the urban population live in slums. [12] Recent national surveys indicate that on average, slum dwellers have poorer health indicators than rural residents. Rates of under-five mortality best exemplify these differences, with 57 child deaths per 1,000 live births reported in urban slums compared to 49/1000 in rural areas. [13] Until recently, an emphasis on maternal and child health characterized Bangladesh’s limited primary care capacity in urban areas, with NGOs and contracted out services providing most of these services. [14] However, with evidence emerging that Non-Communicable Diseases (NCDs) account for over 74% of deaths in low-and middle-income countries [15], attention is shifting. Bangladesh is no exception, with predictions that the prevalence of diabetes will rise to 50% by 2030, making it the 9th ranked country in the world. [16] However, nationally representative data on NCDs is largely confined to studies of risk factors disaggregated by rural/urban, sex and socioeconomic status. [17,18] While comparatively limited data exists on slums [19–22] they point to alarming levels of tobacco use among men, overweight and raised total cholesterol in women, as well as rates of diabetes well above the national average. [17,19,21]

Given the health risks and challenges that slum-living presents, we need to better understand the full range of chronic health problems that slum dwellers face, the healthcare choices they make [22–25] and the economic burden this represents. However, evidence suggests that illness experiences may vary substantially between and within slums due to differences in socio and physical environment, geography, income poverty as well as neighborhood and habitat type [7,21,26,27]. The impact of these characteristics has been noted by Khan (2012) in urban India, where notable inter-slum variations in MNCH practices were observed. [10] Less is known about variations in healthcare seeking for chronic conditions in slum settings, and what evidence exists focuses more on averages than the complexities that determine health-seeking. [22–24]

Given Bangladesh’s fragmented and pluralistic healthcare system, and the dominant presence of the informal private sector in and around slum settlements [28], heterogeneity in disease burden, health seeking behavior and health expenditures are likely. In this study we document this complexity in two slum settings in Bangladesh. We examine age, sex, socioeconomic and geographic patterns of healthcare seeking for reported chronic illness among the working age population (15–64) and consider the catastrophic impact of healthcare spending. Our findings challenge the tendency to view slum populations as homogeneous and point to the need for slum-specific strategies for chronic disease prevention and management.

## Methods

### Operationalizing non-communicable diseases

Current discourse on NCDs tends to focus on four common chronic conditions—cardiovascular disease, cancer, chronic respiratory disease, and diabetes [29,30]—given their substantial contribution to NCD-related morbidity and mortality. [31] Notwithstanding, concentrating only on this cluster of NCDs underestimates the burden of a range of chronic symptoms that impact well-being. Firstly, in observational studies or surveys that report prevalence, due to the costs of NCD diagnosis based on biomedical and cytological specimen collection, many researchers rely on self-reported data [21,32] whereby individuals indicate whether they have been previously diagnosed by a qualified medical professional. This approach will underestimate actual prevalence in slum dwellers where such conditions may remain undiagnosed as appropriate healthcare may not be sought nor available. [19] Secondly, for slum dwellers who work in the informal sector with little employment security, the impact of chronic symptoms of musculoskeletal pain, gastric pain, long-term fever etc. may negatively impact their ability to earn and support their families. Considering these factors, in this study we have chosen to define NCDs in terms of chronic symptoms to capture the full range of ailments that slum dwellers report.

### Study design and setting

This cross-sectional survey was undertaken over the period 2013 to 2014 in slum settlements located in two distinct urban sites selected purposively for their differences in health indicators and economic characteristics. The first site was Sylhet City Corporation, a divisional capital located in the East part of the country, known for its social and religious conservatism. Sylhet Division consistently ranks among poorest performers in terms of health outcomes and utilization of healthcare services in the country [33] and is known to receive among the largest inflows of remittance income in the country. Sylhet City has a population of approximately, 550,000, of which 27% are slum dwellers engaged in day labor and small trading activities. [34,35] The second site is Tongi, a township of about 400,000 population located within the newly formed Gazipur City Corporation. [35] Adjacent to Dhaka (the capital city), Gazipur’s total population of approximately 2.5 million are spread over an area of 330 square kilometres. Tongi is the centre of Gazipur’s export processing zone for ready-made garments and pharmaceutical industries. A large proportion of the population living in Tongi’s sprawling informal settlements are migrants from other parts of the country who have moved into the area for work. Rates of unemployment are low, with men principally employed in service or industrial sectors, and a large proportion of women working in the garment industry.

### Sample

In each site, a 2-stage cluster sampling approach was used to identify households for study inclusion [36]. A list of slum households was provided by a well-known NGO Maternal and Newborn Health program working both sites. Updated on a quarterly basis, the program categorized households according to need using specific targeting criteria. Organizing their program area into geographic zones, community health workers deployed with each assigned to approximately 200 households. For sampling purposes, each 200-household catchment area was considered a cluster. In Sylhet, 30 of 189 clusters were randomly selected, with 10 clusters in each zone. An additional 9 clusters were added during the survey process to reach targeted numbers of participants in certain age groups. In Tongi, 34 of 330 clusters were randomly selected.

The research team visited each household in the selected clusters and recorded household members’ age, sex, experience with illness in the last three months and whether the illness was acute or chronic. The resulting sampling frame for chronic disease was stratified by age and sex, and sample size for each city was determined with reference to existing population health statistics. [37] For the purposes of this analysis, we considered a subset of household members falling into the working age range of 15–64 years who reported chronic illness with a duration of three or more months. Because the sampling was random, it was possible to have respondents of different age and sex groups interviewed within one household.

### Data collection

To enable comparability, the data collection instrument was designed in line with existing health seeking behavior items in the Bangladesh Urban Health Survey, the Bangladesh Demographic and Health Survey, the Household Income and Expenditure Survey [38] and a survey undertaken by Smiling Sun [13, 33, 39].

The Progress of Poverty Index ^®^ (PPI) tool [40] by Grameen Foundation was used to assess HH socio-economic status. The English and Bangla versions of the survey instrument are available in a S1 and S2 Files. All these questionnaires exist in the local language Bangla and have not been validated in our study.

The survey was fielded by 4 teams, each comprised of 8 research assistants and a supervisor with prior experience in survey implementation. Following a 7-day training session, a 2-day pre-test in one of Dhaka’s slums was conducted with ten respondents to refine. Problems around erasures, skipped questions, misread or overlooked instructions, missing answer options, and questions not easily understood were noted and remedied. The finalized questionnaire was was uploaded on a tablet computer. The easy user interface ensured easy data collection and the pre-programmed skip patterns and answer options minimized possibility of errors in data collection. Supervisors also monitored interviews regularly and reviewed tablet entries for errors and inconsistencies. Any issues that arose were resolved by asking the field teams to contact the respondent and clarify details. When respondents were absent, field teams revisited or organized a more convenient time for interview. Once the supervisors were satisfied with the quality and completeness of data collected, data were uploaded into a central server. A data management team reviewed incoming data for consistency and completeness a final time prior to analysis.

The survey for chronic illness asked respondents to indicate their age, sex, occupation and education, and to report the symptoms and severity of any chronic condition they were experiencing. Respondents were also asked where they last sought treatment and why a particular type of provider or facility was chosen. At the household level, expenditure data were elicited according to the typical timeframe of each expenditure i.e. daily for food, weekly for transport and mobile bills, monthly for rent, utilities, and education, or yearly for clothing, furniture, and miscellaneous events such as weddings, health emergencies, then standardized into yearly expenses. Detailed information on regular acute and chronic healthcare expenditures were also elicited, including the costs of drugs, transportation, consultation and other informal payments.

### Data analysis

Data were analyzed using STATA v.15. All analyses were performed using weights that reflected the likelihood of selection at each stage of sampling [36], and all statistics represent weighted data. Missing values were low (<2% for each variable); cases with missing values were not included in analyses.

Weights were calculated for each city separately. The study used a two-stage cluster sampling; sampling weight was calculated based on the sampling probability separately for each sampling stage and cluster. [41] All analyses used the *svy* functions in STATA v. 15, that adjust for complex survey sampling. [42]

#### Logistic regression

Multinomial logistic regression was used to predict the odds of choosing a particular type of health care facility for the most recent (adult) chronic care visit considering the independent variables; sex, age, city, and PPI score [41]. Independent variables were analyzed between subjects. Models with different base categories are presented so all pairwise comparisons can be seen. Significance levels were set at .05.

#### Variable definitions

*Catastrophic health expenditures*. The incidence and intensity of catastrophic health expenditures were calculated for all types of illness at household level using methods explained by the World Bank. [43] Incidence is the estimated proportion of the sample whose health care costs as a share of total (or nonfood) expenditures exceeds a certain threshold, referred to as Head Count. Intensity is the average amount by which households exceed the threshold and is referred to as Overshoot. Incidence and intensity are both reflected in Mean Positive Overshoot, which is Overshoot divided by Head Count. Weighted Head Counts and Overshoots take into account the distribution of expenditures by relative wealth by applying concentration indices. [43] Results are presented in BDT (Bangladeshi Taka) whereby approximately 75–85 taka are equivalent 1 USD.

*Chronic disease category*. Diseases were classified using the International Classification of Primary Care, 2nd edition (ICPC2) codes by trained medical doctors. For the purposes of analyses, the following chronic disease categories were used: Cardiovascular, Digestive, Endocrine, Musculoskeletal, Neurological, and Respiratory. These categories encompassed the most frequently cited chronic conditions as well as those (such as diabetes under Endocrine) that are of increasing concern. All other categories were grouped together as “other”.

*Health care facility*. The most commonly used healthcare facilities for chronic care visits were drug shops (most lacking a qualified pharmacist), government hospitals, private hospitals or clinics, and doctor’s chambers. The percentage of most recent visits for chronic health conditions to one of these four facilities was 93.1% in Sylhet and 88.5% in Tongi. Our analyses, therefore, focused on comparing these four types of healthcare facilities.

*Progress out of Poverty Index (PPI)*. PPI scores were used as a proxy for socioeconomic status. PPI is comprised of 10 nonfinancial high poverty correlated indicators that are simple and verifiable. [40] These indicators include the number of children in the family, the number of children attending school, household members’ employment, size of house, type of house construction materials, and ownership of televisions, fans, mobile phones, bicycles, motorcycles, scooters or cars, and cultivable agricultural land. Scores range from 0 (poor) to 100 (not poor) and can be converted to country-specific risk of falling below the poverty line. Both raw PPI scores and PPI quintiles were used in analyses. PPI quintiles were created separately for Sylhet and Tongi using weighted data.

*Occupation*. Occupations were grouped based on similarities in levels of autonomy, type of work, and household PPI score etc. The following categories were thus identified: Day Laborer/Rickshaw Puller, Security Guard/Factory Worker/Service, Small business/Motorized Transport Driver, Skilled Laborer/Sales, Housewife/Unemployed, Business, and Other.

### Ethics approval

The proposal and survey tools were reviewed and approved by the Research Review Committee and Ethical Review Committee of icddr,b. Before starting each interview, written consent was obtained from each survey respondent. All elements of informed consent were presented orally in advance of signing to ensure that each respondent understood the purpose of the research, the measures undertaken to ensure confidentiality, and their right to withdraw from the interview at any time, for any reason. Protocols were observed to ensure respondent confidentiality, privacy and data protection.

## Results

### Background characteristics of study participants

A total of 1,045 participants were surveyed, with an even distribution of respondents between the two slum sites. Table 1 presents the demographic and economic profile of respondents in Tongi and Sylhet. In Sylhet, males comprised 45% of all adult respondents and females 55%. This proportion was reversed in Tongi, with 56% male and 44% female. The mean age of respondents was comparable at approximately 41 years in both sites, with 80% identifying as married at the time of the survey. Tongi’s average PPI score of 57 was higher than the Sylhet average of 49. The breakdown of PPI quintiles in Table 1 displays the same trend.

**Table 1:** Sample characteristics of working age adults (15–64) (weighted percentages and means).

	Sylhet (N = 509)	Tongi (N = 536)
Male, %	45.34	56.18
Female, %	54.66	43.82
Age, Mean (SE)	41.29 (0.84)	41.13 (0.79)
Marital Status		
Married, %	76.95	78.41
Unmarried, %	9.47	9.79
Widowed, %	11.78	10.80
Separated/Divorced, %	1.80	1.00
PPI Score, Mean (SE)	49.03 (0.92)	57.48 (0.71)
PPI Quintile		
1, %	13.48	19.36
2, %	21.44	15.29
3, %	16.28	17.15
4, %	22.58	22.08
5, %	26.21	26.12

Table 2 presents the occupational characteristics of survey respondents. Overall, 47.2% of all participants in Sylhet and 36.2% in Tongi reported being unemployed or not working for pay. Women identifying as housewives comprised the largest proportion of this group in both study sites. Study respondents in Tongi engaged in regularly paid jobs such as night guard, any service, and factory work in a greater proportion (37.9%) than those in Sylhet (17.5%). By contrast, a higher proportion of respondents in Sylhet were engaged in small business or motorized transport compared to Tongi (10% vs. 6% respectively).

**Table 2:** Occupational distribution of working age adults (15–64 years) by sex (weighted percentages).

	Sylhet (N = 11,888)			Tongi (N = 13,959)		
	*Male*	*Female*	*Total*	*Male*	*Female*	*Total*
	*%*	*%*	*%*	*%*	*%*	*%*
Day labor/Rickshaw puller	17.83	1.99	9.24	11.75	0.25	6.65
Guard/Service/Factory	20.64	14.94	17.55	43.03	31.38	37.86
Small business/Motorized transport	20.00	1.60	10.02	9.59	1.88	6.17
Skilled labor/Sales	13.70	0.62	6.61	6.23	1.34	4.06
Housewife/Unemployed	11.73	77.19	47.22	15.70	61.94	36.21
Business	12.02	1.50	6.32	9.31	0.44	5.38
Other^a^	4.08	2.16	3.04	4.40	2.76	3.68

^a^Includes student.

### Chronic Illness and healthcare seeking characteristic by household SES

The prevalence of chronic illness in adults of working age (15–64) across randomly selected households located in poor informal settlements was 40.6% for Tongi and 34.3% for Sylhet. Those households with adults reporting chronic illness constitute the sample on which all subsequent analyses are performed.

In both sites, socioeconomic status varied in a similar pattern across the illness symptoms for which treatment was last sought (Table 3). Those seeking care for respiratory, digestive, musculoskeletal, and neurological symptoms had lower PPI scores while those seeking care for cardiovascular and endocrine symptoms had higher average PPI scores—above 54 in Sylhet and above 60 in Tongi.

**Table 3:** Mean PPI score by most severe symptom for which treatment was last sought.

Most severe symptom	Sylhet			Tongi		
	*M*	*SE*	95% CI	*M*	*SE*	95% CI
Cardiovascular	54.49	2.50	[49.43, 50.66]	60.67	2.50	[55.59, 65.75]
Digestive	47.58	2.18	[43,16, 52.00]	57.62	1.43	[54.71, 60.52]
Endocrine	59.23	4.33	[50.46, 67.99]	64.77	3.82	[56.99, 72.54]
Musculoskeletal	46.46	2.08	[42.26, 50.66]	55.84	1.64	[52.50, 59.18]
Neurological	52.51	2.52	[47.41, 57.60]	58.82	1.72	[55.32, 62.32]
Respiratory	48.99	2.50	[43.93, 54.04]	57.92	1.87	[54.11, 61.74]

SE = Standard Error

**Fig 1:**
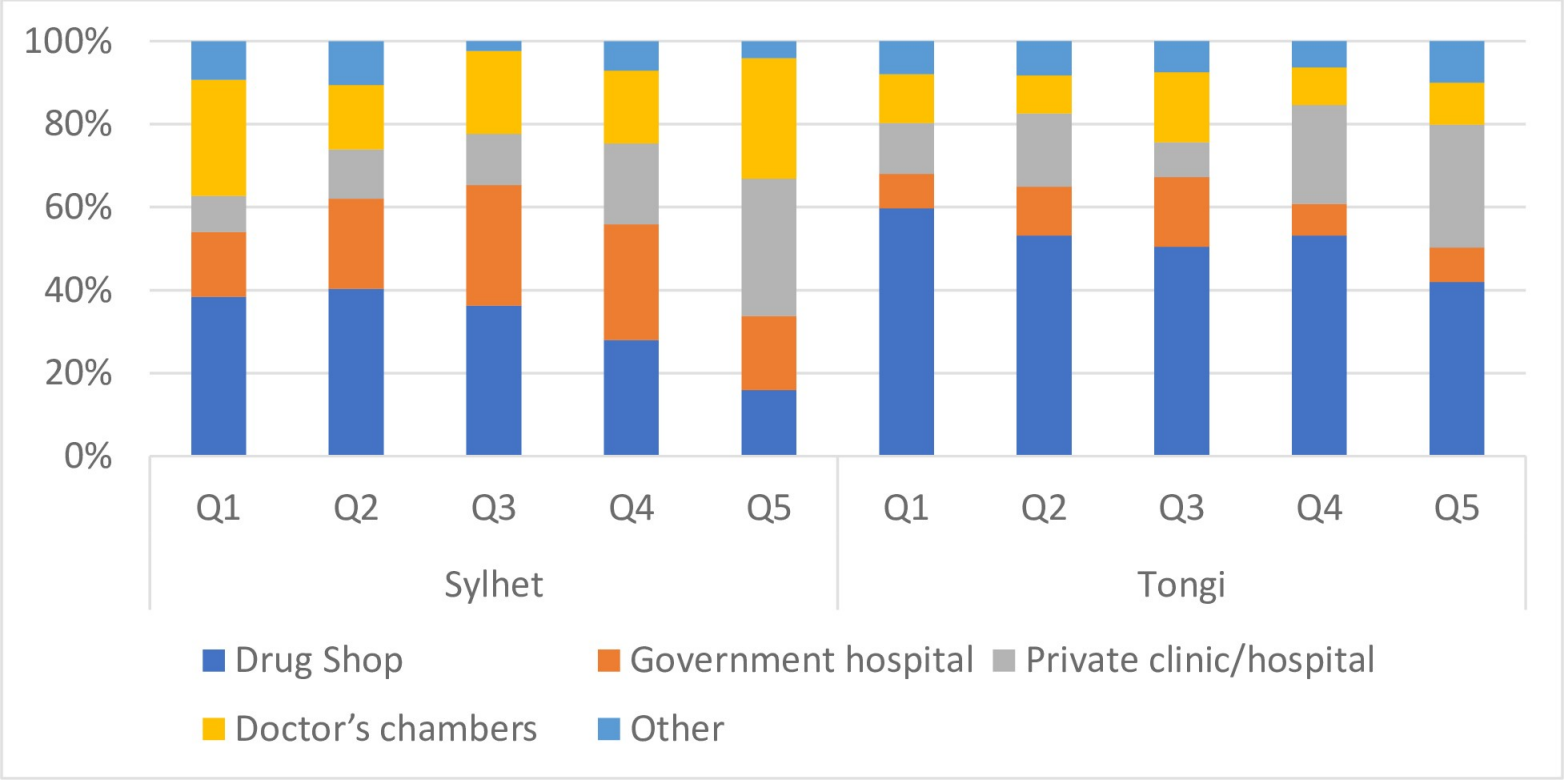
Type of facility for which care was last sought for chronic condition by PPI quintile and city.

Fig 1 displays the type of facility from which healthcare was sought the last time. As might be expected, in Sylhet, those in the lowest quintiles sought healthcare from drug shops the most. This proportion fell from 38.5% to 16% from quintiles 1 to 5. A similar descending pattern is found for government hospitals with the wealthiest quintile (Q5) utilizing them least (15.6%). In Q5, the large proportion of healthcare is sought from private-for-profit sector doctors’ chambers and clinics. This pattern is also apparent in quintile 4, but with a comparatively larger proportion of this quintile seeking care from drug shops.

Results from Tongi are very different. Across all quintiles drug shops were the most utilized type of health facility: Q1-59.8%, Q2-53.2%, Q3-50.5%, Q4-53.1%, Q5-42.0%. The next most frequented health facility were private clinics in quintiles 2, 4 and 5, and doctors’ chambers in first and third quintiles.

**Fig 2:**
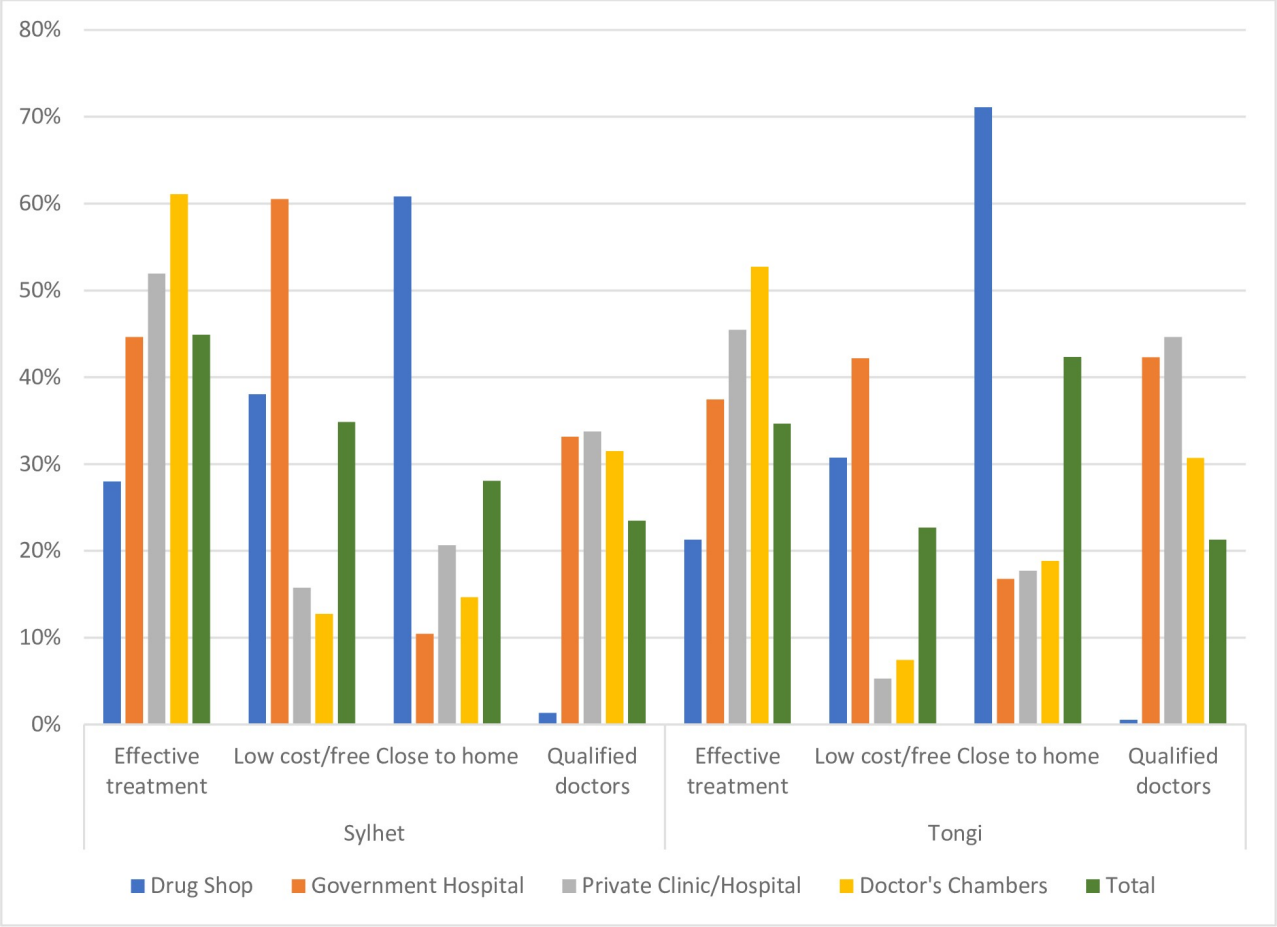
Top four reasons for going to specific providers, by city. Percentages add up to more than 100 because multiple responses were allowed.

The top four reasons for choosing a specific type of provider for the last healthcare visit were effective treatment, low cost or free treatment, proximity to residence, and the presence of qualified doctors (Fig 2). Respondents in Sylhet prioritized effective treatment the most often, and among those doing so, 61% said they went to a doctor’s chamber and 52% to private clinics for that reason. Low cost or free treatment was the second most common reason for seeking care from a particular facility. Reported by one-third of respondents, the availability of low-cost treatment was cited by 60.5% of respondents as prompting their use of government hospitals, followed by 38% who utilized local drug shops for this reason.

In Tongi, proximity was the most important criteria guiding choice of last health provider for 42.3% of respondents. Nearly three-fourths of these respondents indicated the proximity of drug shops was the main factor explaining their choice. Effective treatment was identified by 52.7% of respondents as justification for choosing doctors’ chambers, while 45.5% of respondents chose private clinics for this reason.

For both sites, the presence of qualified doctors was the least mentioned attribute that influenced choice of healthcare facility.

### Determinants of healthcare provider choice

Table 4 displays results for a multinomial logistic regression that predicts the choice of health care provider among the working age population (15–64) seeking treatment for chronic illness by age, sex, PPI score and site. In this table, each independent variable is examined by a base provider group relative to alternate providers to enable easier comparison among healthcare provider types. Logistic regression results indicate that choice of healthcare provider is associated with sex, city, and PPI Score. Age was not significantly related to the choice of provider. Women are almost two times (RRR 1.92, p<0.006) more likely than men to seek care from private clinics as opposed to drug shops. Women also are more likely to seek care from private providers than from government hospitals (RRR 1.82, p<0.028) than are men. Compared to Tongi, respondents in Sylhet slums were approximately 4 times more likely to go to a government hospital or private doctor than drug shops, and 2 times more likely to attend a private clinic than drug shops. Finally, as PPI score increased, the likelihood of going to providers other than drug shops increased, with the biggest increase for private clinics. For each one-point increase in PPI score, there was a 4% increase in the likelihood of going to a private clinic rather than a drug shop.

**Table 4:** Multinomial logistic regression of factors associated with choice of health care provider for chronic illness among adults (15–64), n = 1883.

Health facility	M1. Drug shop base outcome			M2. Government hospital base outcome			M3. Private clinic/hospital base outcome		
	RRR	P value	95% CI	RRR	P value	95% CI	RRR	P value	95% CI
Drug shop	Base Outcome								
sex (ref male)				0.94	.812	[0.59, 1.52]	0.52	**.006**	[0.33, 0.82]
age				0.99	.310	[0.98, 1.01]	0.99	.077	[0.97, 1.00]
City (ref Tongi)				0.23	**< .001**	[0.13, 0.39]	0.47	**.003**	[0.29, 0.76]
PPI score				0.99	.055	[0.97, 1.00]	0.96	**< .001**	[0.94, 0.97]
intercept				16.76	< .001	[5.38, 52.21]	71.28	< .001	[22.30, 227.77]
Government hospital				Base Outcome					
sex (ref male)	1.06	.812	[0.66, 1.70]				0.55	**.028**	[0.32, 0.93]
age	1.01	.310	[0.99, 1.02]				1.00	.607	[0.98, 1.01]
City (ref Tongi)	4.38	**< .001**	[2.55, 7.52]				2.04	**.013**	[1.17, 3.58]
PPI score	1.01	.055	[1.00, 1.03]				0.97	**.003**	[0.96, 0.99]
intercept	0.06	< .001	[0.02, 0.19]				4.25	.037	[1.09, 16.57]
Private clinic hospital							Base Outcome		
sex (ref male)	1.92	**.006**	[1.22, 3.03]	1.82	**.028**	[1.07, 3.08]			
age	1.01	.077	[1.00, 1.03]	1.00	.607	[0.99, 1.02]			
City (ref Tongi)	2.14	**.003**	[1.31, 3.51]	0.49	**.013**	[0.28, 0.86]			
PPI score	1.04	**< .001**	[1.03, 1.06]	1.03	**.003**	[1.01, 1.05]			
intercept	.014	< .001	[0.00, 0.04]	0.24	.037	[0.06, 0.92]			
Doctor’s chamber									
sex (ref male)	1.32	.246	[0.82, 2.13]	1.25	.415	[.073, 2.15]	0.69	.165	[0.41, 1.17]
age	1.01	.369	[0.99, 1.02]	1.00	.810	[0.98, 1.01]	0.99	.395	[0.98, 1.01]
City (ref Tongi)	3.71	**< .001**	[2.14, 6.45]	0.85	.595	[0.46, 1.57]	1.73	.059	[0.98, 3.07]
PPI score	1.02	.052	[1.00, 1.03]	1.00	.872	[0.98, 1.02]	0.97	**.006**	[0.96, 0.99]
intercept	0.06	< .001	[0.02, 0.20]	1.03	.964	[0.27, 3.95]	4.38	.036	[1.11, 17.34]
Other									
sex (ref male)	1.41	.252	[0.78, 2.57]	1.34	.387	[0.69, 2.60]	0.74	.354	[0.38, 1.42]
age	1.01	.124	[1.00, 1.03]	1.01	.565	[0.99, 1.03]	1.00	.868	[0.98, 1.02]
City (ref Tongi)	1.43	.262	[0.76, 2.71]	0.33	**.002**	[0.16, 0.66]	0.67	.232	[0.34, 1.30]
PPI score	1.01	.527	[0.99, 1.03]	0.99	.518	[0.97, 1.02]	0.97	**.005**	[0.94, 0.99]
intercept	0.05	< .001	[0.01, 0.19]	0.86	.842	[0.19, 3.96]	3.65	.103	[0.76, 17.43]

### Catastrophic health expenditure

As displayed in Table 5, the mean annual healthcare spending in Tongi (BDT 27,300 or USD 323) was nearly two-fold more than Sylhet (BDT 14,246 or USD 169). In Sylhet, the highest expenditure was made by households in the fourth quintile at BDT 18,902 (~224 USD) and the differences between the quintiles varied by 2,000–3,000 BDT (~23–35 USD). In Tongi, quintiles 1 to 4 spent below BDT 28,000 (~USD 333), while the wealthiest households in quintile 5 spent almost 50% more at BDT 40,789 (~USD 483) than the poorest households (Q1). However, when we annualize the costs of healthcare for chronic illness for each respondent in our sample, we see that mean overall spending is slightly lower for Tongi (5,304 BDT) than for Sylhet (6,305 BDT).

**Table 5:** Household Annual Healthcare Spending by PPI quintile in BDT (weighted means).

	Household Annual Expenditure	
	Sylhet (N = 25,645)	Tongi (N = 37,882)
	*M (SE)*	*M (SE)*
PPI Quintile		
1	9,657 (1,491)	19,026 (4938)
2	12,119 (1,659)	26,858 (6,666)
3	14,092 (2,882)	19,743 (3,443)
4	18,902 (2,541)	25,244(7,736)
5	15,750 (2,028)	40,789 (7,602)
Total	14,246 (961)	27,300 (2,936)

Table 6 shows the incidence and intensity of household-level catastrophic health expenditures. Poor households in Tongi have slightly higher catastrophic health expenditures (CHE) compared to Sylhet. Out-Of-Pocket (OOP) healthcare-related payments over 10% of total household expenditure were reported by 28% of households in Tongi and 21% households in Sylhet. Also, in Tongi the weighted head count is slightly lower than the unweighted indicating that the richer households tended to exceed the 10% payment threshold more than the poorer households. This was not apparent in Sylhet.

**Table 6:** Incidence and intensity of catastrophic healthcare payments defined with respect to total and nonfood expenditures, various thresholds.

*Catastrophic payments measures*	*Threshold budget share*					
*Healthcare spending as share of total expenditure*	*Sylhet*			*Tongi*		
	*10%*	*15%*	*25%*	*10%*	*15%*	*25%*
**Head Count**^1^						
1 (Poorest)	20.97	11.31	1.85	19.67	12.48	7.04
2	16.79	9.47	4.80	32.32	19.09	12.89
3	16.17	9.40	0.84	27.05	15.11	7.92
4	29.48	20.67	2.68	22.73	9.37	4.53
5 (Richest)	23.88	16.61	2.45	37.06	21.16	12.41
Total	20.75	13.35	2.48	28.18	15.40	8.98
**Weighted Total Head Count**	20.49	13.03	2.65	26.20	14.57	8.26
**Overshoot**^2^						
1 (Poorest)	1.62	0.71	0.05	2.61	1.81	0.96
2	1.78	1.11	0.31	4.91	3.70	2.10
3	1.17	0.56	0.04	3.00	1.94	0.97
4	2.48	1.20	0.22	1.99	1.30	0.76
5 (Richest)	1.88	0.92	0.14	4.87	3.40	1.79
Total	1.75	0.89	0.16	3.46	2.41	1.30
**Weighted Overshoot**	1.73	0.89	0.16	3.32	2.36	1.31
**Mean Positive Overshoot**^3^	**8.42**	**6.65**	**6.30**	**15.54**	**15.43**	**19.28**
**Concentration Index, C_E**^4^	0.012	0.024	-0.066	0.070	0.054	0.080
**Concentration Index, C_O**^4^	0.009	-0.000	-0.042	0.040	0.023	-0.009
*As share of nonfood expenditure*	*15%*	*25%*	*40%*	*15%*	*25%*	*40%*
**Head Count**^1^						
1 (Poorest)	42.97	15.08	5.31	39.29	16.93	7.05
2	34.92	10.22	5.43	44.40	22.94	15.47
3	24.91	13.10	1.67	30.67	18.57	9.55
4	40.01	21.67	5.44	38.02	10.19	5.16
5 (Richest)	29.57	19.46	4.43	44.85	22.88	11.87
Total	32.50	15.60	4.42	39.95	18.38	9.67
**Weighted Total Head Count**	34.72	15.36	4.82	40.68	18.67	9.43
**Overshoot**^2^						
1 (Poorest)	5.36	2.41	0.86	5.20	2.66	1.09
2	3.90	1.76	0.53	8.86	5.86	3.07
3	2.86	0.97	0.14	5.26	3.10	1.19
4	5.58	2.67	0.48	4.00	1.94	1.11
5 (Richest)	4.13	1.82	0.35	7.55	4.39	1.86
Total	4.20	1.89	0.45	6.17	3.54	1.64
**Weighted Overshoot**	4.39	1.94	0.49	5.98	3.40	1.56
**Mean Positive Overshoot**^3^	**12.91**	**12.09**	**10.29**	**15.44**	**19.29**	**16.98**
**Concentration Index, C_E**^4^	-0.068	0.015	-0.091	-0.001	0.021	0.066
**Concentration Index, C_O**^4^	-0.046	-0.028	-0.077	0.030	0.041	0.048

^1^Percentage of households with adults with chronic illnesses who exceed the threshold for share of budget spent on health care

^2.^ Average percentage of expenditures over the threshold spent on health care

^3.^Overshoot/Percent above threshold

^4^Concentration Index is a measure of socioeconomic inequality in a health measure. A value of 0 indicates no inequality, while negative values indicate disproportionate concentration among the poorer groups while positive values indicate disproportionate concentration among the wealthier groups. C_E indicates concentration index for expenditures; C_O indicates concentration index for Overshoot.

When considering percentage of total non-food expenditures, twice as many households in Tongi (9.67%) spend more than 40% of total non-food expenditures on medical expenses than households in Sylhet (4.4%). Although only a small difference, it was seen that the poorer households in Sylhet exceeded the payment threshold in the non-food expenditure category more than the richer households, i.e. the weighed head count at the 40% threshold was 4.8% which is higher than the unweighted of 4.4%. A reverse scenario was evident in Tongi. The proportion of households with total OOP payments above 40% of their total non-food expenditure was 9.7%. This proportion decreases to 9.4% after applying weights to different socio-economic groups.

The mean positive overshoot represents the extent to which household health payments exceed various thresholds on average. Approximately 8% and 15% of households in Sylhet and Tongi respectively had OOP health expenditures exceeding 10% of their total budget. The corresponding value for non-food budget share above 40% was 10.3% in Sylhet and 17% in Tongi.

There is no clear pattern of OOP expenditures by wealth quintile (measured by PPI score), although it appears that wealthier quintiles tended to spend a greater percentage of their total budget than poorer quintiles in Tongi. This is borne out by positive (albeit very small) concentration indices (Table 5). Poorer households in Sylhet tended to spend a greater percentage of their nonfood expenditures, however, on healthcare expenses, as seen by the mostly negative concentration indices (CIs). Again, the CIs were very small, reflecting the fact that the sample was restricted to low-income residents, and was therefore not reflective of the income distribution of the total population in these cities.

## Discussion

### Between and within slum differences

Study results reveal the highly heterogeneous nature of slum settings. While sharing the typical attributes of low-income settlements, such as inadequate sanitation and poor housing durability [44], the two study sites differed in terms of average wealth score (PPI) and occupational profile. Recognizing that a PPI score below 80 is considered poor, Tongi’s average score was substantially higher than Sylhet (57 vs 49 respectively). Underlying some of this difference is the greater availability and higher prevalence of more secure factory-based employment in Tongi compared to Sylhet, where the proportion of slum dwellers involved in informal day labour and business is much higher. [35]

In terms of reported health status, a high prevalence of adult chronic illness was found in slum settlements in Tongi (41%) and Sylhet (34%), most likely reflecting the adverse environmental conditions and/or work-related stressors and exposures that slum dwellers experience. Distinctive socioeconomic patterns in chronic symptoms were also observed with lower mean PPI scores for musculoskeletal/digestive symptoms and higher scores for reported endocrine and cardiovascular symptoms. While there is scant literature that examines wealth-related differentials in chronic illness symptoms within or between slums [24], study results are consistent with the literature documenting population level socioeconomic inequalities in specific NCDs, both in Bangladesh [45] and globally [46,47]. Most similar to our findings, is a study in rural Tamil Nadu which found that individuals in poorer households were more likely to report connective tissue problems related to heavy manual labour, than sedentary higher caste households where diabetes and circulatory diseases were more prevalent. [48] As noted by Dodd et al. (2016), the under-diagnosis of endocrine and cardiovascular disorders is expected in resource constrained settings, as is the tendency to report on chronic ailments that impair productivity especially among those whose livelihood is day to day. [46]

### Healthcare seeking for chronic illness

A second major finding concerns the widespread use of informal providers among slum dwellers in both study sites. Although residents of poor urban settlements were relatively better-off in Tongi, they relied to a greater extent on drug shops and pharmacies, and cited reasons of proximity for this choice, followed by the availability of effective treatment. Open during evening hours, these facilities accommodate the shift schedules of factory workers and offer the convenience of “quick-fix medicine” to minimize days off work. Similar patterns of health seeking are reported in small-scale studies of the urban poor in Sri Lanka [49] where the convenience of pharmacies was preferred over the long queues and wait times associated public providers.

In Sylhet, nearby access to government hospitals and the greater availability of private doctor’s chambers, many of whom are dual practitioners working in the public sector, may account for the comparatively greater use of qualified public and private sector services than in Tongi. In both slum settings, greater wealth increased the likelihood of going to providers other than drug shops, with the biggest increase for private clinics. These findings are consistent with findings from Karachi’s slums, where higher income slum residents reported a similar reliance on private sector clinics. [25] Naydenova (2018) reported similar findings in their study of slums in Mumbai, noting an overall preference for private sector care, and an association between socioeconomic status and choice of healthcare provider. [23]

Sex differences in chronic healthcare seeking were also apparent, with women across slums relying on private clinics to a greater degree than men compared to drug shops or public facilities. These findings may be related to the attributes slum dwellers value in making healthcare provider choices for chronic illness care. For women, the perception that private clinics offer effective care might encompass issues of respect and privacy, which informal and public sources of care may be less able to ensure.

### Catastrophic healthcare expenditure

Out-of-pocket expenditures in Bangladesh constitute about 70% of total expenditure on health [50] and rates of catastrophic health expenditure (CHE) are among the highest in the South Asian region. [51] Most calculations of CHE are based on city or national-level data sets [52,53], making this cross-sectional study one of the first to examine CHE in the context of two distinctive slum settings. Our findings revealed high and variable levels of overall healthcare spending, with households in Tongi spending nearly twice as much on annual healthcare than Sylhet, and the highest quintile spending two times more than the poorest. When we consider rates of catastrophic health expenditures (CHE), differences between slums are reduced, ranging between a weighted headcount of 21–28% of total household spending (over a 10% threshold), although weighted head counts indicated a slightly greater propensity to exceed this threshold among rich households in Tongi. Compared to national-level estimates of 16.3% in rural areas and 10.6% in the lowest wealth quintile in urban areas [53], our findings emphasize the value of more textured studies of CHE given the socioeconomic diversity of slum residents and concentrated health risks they face.

At a threshold of 40% of total non-food expenditure [53], Khan et al. (2017) found an average CHE headcount of 11.6% in rural households and 9.8% in the poorest quintile in urban areas, compared to our estimates of 9.67% and 4.4% in slum settlements in Tongi and Sylhet respectively. We also calculated weighted headcounts indicating a slightly greater CHE in richer households in Tongi, and among poorer households in Sylhet. Explanations for these variable patterns may relate to differences in the availability of publicly provided healthcare between sites, and the omission of those so poor that they fail to seek care for illness. In short, results emphasize that the opportunity costs of illness among slum dwellers is substantial, but that huge heterogeneity exists in their ability to spend on healthcare. Not captured in these figures, however, are individuals who fail to seek care for illness due to poverty or choice, nor the productivity costs of chronic illness and its impact household livelihood.

### Limitations

Given the complex and dynamic nature of slum settings, study findings should not be generalized. There is also risk of error and recall bias associated with self-reported symptoms of chronic illness. It is likely, however, that recall accuracy is comparatively better for chronic vs. acute illness events given their duration of 3 or more months. To further minimize recall bias, we focused on care received at the last visit, and not the first which may have occurred many years prior. A possible consequence of using this approach is that the respondent’s primary choice of provider is not recorded as health-seeking choices may have varied over subsequent visits.

## Conclusions

The global Sustainable Development agenda recognizes urban slums as a priority focus in achieving SDGs 3 and 11 given their growing presence in LMIC cities, and the health threats they pose in the absence of investments in basic services, infrastructure and social and economic opportunity.

This study depicts a complex landscape of chronic health challenges and healthcare choices that slum dwellers must navigate. Striking heterogeneity is revealed that counter simplistic notions that slum dwellers are uniformly poor. However, good health is similarly valued and no matter how poor, the large majority seek healthcare in whatever form they can access depending on the realities of proximity, time, perceived effectiveness and cost. Understanding these context-realities will be important when designing delivery and financial protection strategies that address the growing burden of NCDs in slum settings and the impoverishing effects of catastrophic healthcare spending.

The study also points to the value of broader definitions of NCDs beyond a concern for cardiovascular, cancer, respiratory disease and diabetes, and which encompass chronic conditions that impact health and productivity. Recognizing the disabling burden of widespread musculoskeletal, respiratory and other ailments, efforts are needed to improve working and living conditions that give rise to chronic symptoms, and to develop responsive health systems including integrated care models, and greater provider capacity around prevention and treatment.

The prominence of informal sources of care like drug shops, as sources of healthcare and medicine that are proximate and trusted, is another issue of concern. Implementation research is needed which investigates how best to create demand for qualified healthcare, and how to design healthcare delivery systems that address issues of convenience and affordability that are valued by the working poor.

Finally, in the absence of strong systems of public primary healthcare in urban Bangladesh, the reality of the private sector must be addressed. To achieve Universal Health Coverage in urban areas, it is imperative that this sector be systematically integrated in health systems strategies, that regulatory systems be strengthened, and that financial protection mechanisms be enacted to protect slum and other vulnerable households from medical impoverishment.

## Supporting information

S1 File Survey instrument in Bangla. (PDF)

S2 File Survey instrument in English. (PDF)
