# Supplementary material for: Healthcare seeking for chronic illness among adult slum dwellers in Bangladesh: A descriptive cross-sectional study in two urban settings
Source: PLoS One. 2020 Jun 15;15(6):e0233635. doi: 10.1371/journal.pone.0233635 (PMC7295220; doi:10.1371/journal.pone.0233635)
Supplement: S1 File — (PDF) [file pone.0233635.s001.pdf]

Healthcare Seeking Behavior among the poor in urban areas of Bangladesh, 2013

<table>
<tr><th colspan="3">HOUSEHOLD IDENTIFICATION</th></tr>
<tr><td colspan="2">City Corporation:…………………………………………………
Cluster Number..
Household Number..
Type of Household: 1. Family 2. Mess
Name of The Household Head…………………………………………..
Name of The Respondent…………………………………………</td><td>Household Gps Location:
___ ____ ____ ___.____ ____</td></tr>
<tr><td colspan="3">INTERVIEW DETAILS</td></tr>
<tr><td colspan="2">Interviewer Code:
Result
1st Visits: ___/____/______ Start Time: ___:____ End Time:_____:____
2nd Visits:____/______/____ Start Time: ___:____ End Time:_____:____
3rd Visits:____/______/____ Start Time: ___:____ End Time:_____:____</td><td>Interview Completed
1. Yes
2. No</td></tr>
<tr><td>Cross Checker Name:</td><td colspan="2">Date of Cross Check: ___/______/_______</td></tr>
<tr><td>Supervisor: _____________________
Date: _____/______/________</td><td>Field Editor:_________________
Date: _____/______/________</td><td>Remarks</td></tr>
</table>

সরবরাহকৃত খানার তালিকায় খানার জরিপের ফলাফল নির্দেশ করুন

Healthcare Seeking Behavior Among the Urban Poor in Bangladesh, 2013
Screening Questionnaire for Household Membership Listing

*Eligibility Criteria (Code):

1= Individual Respondent Module (For All)
2= Health Care Seeking Behavior Module (15 yr and Above)
3= Not Eligible

SL	101	102	103	104	105	106	106a	107	107a	107b	107b_1	107c	107c_1	107d	107e	107f	107g	107h	107i	107j
	Name	Sex	Age	Marital Status	Relation to the Household Head**	Mother who has delivered baby within last 1 Year	Are you currently pregnant	Mother/Guardianwho has living child age under 5 years	Mother/Guardian who has living child age between 5-14 years	Health problem for last 14 days	Specify the Disease	Health problem for last 3 months or more	Specify the Disease	Any physical/ mental disability	If yes, have you sought treatment in last 3 months?	Unable speak or hear?	Eligibility for Module *	Available in HH for next 2 weeks?	Available Appointment Time	Eligib e for modu e*
		1=M<br>2=F	-----/-----<br>y/m	1. Married<br>2.Separated<br>3. Divorced<br>4.Widowed<br>5.Unmarried	*See the options below the table***	1=Yes<br>2=No<br>3= NA	1=Yes<br>2=No	1=Yes<br>2=No<br>3= NA	1=Yes<br>2=No<br>3= NA	1=Yes<br>2=No<br>3= NA		1=Yes<br>2=No<br>3= NA		1=Yes<br>2=No	1=Yes<br>2=No	1=Yes<br>2=No	*See the Codes above**			
1.																				
2.																				
3.																				
4.																				
5.																				
6.																				
7.																				
8.																				
9.																				
10.																				

**01 = Head, 02 = Spouse, 03 = Son /Daughter, 04 = Son-in-law/ Daughter-in-law, 05 = Grandchild, 06 = Parent, 07 = Parent-in-law, 08 = Sibling-in-law, 09 = Other relative, 10 =Adopted/ foster/ stepchild, 11 = not related12=housekeeper, 13= Brother/Sister 77 = DK

CHARACTERSTICS OF SOCIOECONOMY<br>DO NOT READ THE OPTION OF ANSWER UNLESS THERE IS A SPECIFIC INSTRUCTION				
	Indicator	Value	Points	Score
108	খানায় ১২ বছর বা তার নিচের বয়সের সদস্য সংখ্যা কত?	A.তিন বা তার বেশী<br>B.দুই<br>C.এক<br>D.কোনটিই নয়	0<br>10<br>16<br>29	
109	আপনার পরিবারের ৬ থেকে ১২ বছরের সকল সদস্য কি বর্তমানে বিদ্যালয়ে বা শিক্ষা প্রতিষ্ঠানে যায়?	A. না<br>B. ৬ থেকে ১২ বছরের কেউ নাই<br>C. হ্যা	0<br>3<br>6	
110	গত এক বছরে আপনার পরিবারের কোন সদস্য কি কখনও দৈনিক মজুরির ভিত্তিতে কাজ করেছেন?	A. হ্যাঁ<br>B. না	0<br>8	
111	আপনার পরিবারে কতগুলো কক্ষ আছে? (ব্যবসার উদ্দেশ্যে ব্যবহৃত কক্ষ বাদ দিয়ে)	A. এক<br>B. দুই<br>C. তিন বা অধিক	0<br>3<br>5	
112	বাড়ির প্রধান কক্ষের দেয়াল নির্মানে ব্যবহৃত প্রধান উপাদান কি?	A. খড়/বাঁশ, বা অন্যান্য<br>B. টিন, কাঠ,<br>C. ইট / সিমেন্ট/ কক্রিট	0<br>2<br>9	
113	আপনার পরিবারে কোন টেলিভিশন আছে?	A. না<br>B. হ্যাঁ	0<br>7	
114	আপনার পরিবারে কতগুলো বৈদ্যুতিক পাখা আছে?	A. একটিও না<br>B. এক<br>C. দুই বা অধিক	0<br>4<br>7	
115	আপনার পরিবারে কতগুলো নিজস্ব মোবাইল ফোন আছে?	A. একটিও না<br>B. এক<br>C. দুই বা অধিক	0<br>8<br>15	
116	পরিবারে কোন নিজস্ব বাই-সাইকেল/ মোটর/ সাইকেল/রিকশা/ভ্যান/ স্কুটার অথবা মোটর কার আছে কি?	A. না<br>B. হ্যাঁ	0<br>4	
117	আপনার পরিবারে কি নিজস্ব (যেমন ভাড়া, ভাগের ফসল,বন্ধক,) ৫১ বা তার অধিক ডেসিমেল চাষযোগ্য কৃষিজমি আছে? (অনাবাদী জমি , নিজ বাড়ি ও উঠান বাধ দিয়ে)	A. না<br>B. হ্যাঁ	0<br>7	

বিঃদ্রঃ পিপি আই খুজে পি পি আই এর স্কোরবোর্ড থেকে মোট স্কোর ব্যবহার করুন।

118 was deleted

TICK HERE IF CONTINUATION SHEET USED	
Just to make sure that I have a complete listing:	
Are there any other persons such as small children or infants that we have not listed?<br>(If YES Go back to household schedule and enter new members in the household schedule.)	
In addition, are there any other people who may not be members of your family, such as domestic servants, lodgers or friends who usually live here?<br>(If YES Go back to household schedule and enter new members in the household schedule.)	

Sl	Questions	Code			Sl
119	আপনার থানায় কি কি আছে ?<br>(বর্তমানে যেখানে বসবাসরত)<br><br>(পড়ে শোনান)		হ্যাঁ	না	
		বিদ্যুৎ	1	2	
		সচল রেডিও	1	2	
		সচল টেলিভিশন	1	2	
		মোবাইল ফোন	1	2	
		রেফ্রিজারেটর/ ফ্রিজ	1	2	
		সাইকেল	1	2	
		মটর সাইকেল	1	2	
		গাড়ি অথবা ট্রাক	1	2	
		রিক্সা /ভ্যান	1	2	
		DVD প্লেয়ার	1	2	

Household member, occupation and income<br>*If mess- only record individual's income*							
Line No	101	120a	120b	120c	120d	120f	120g and h
	নাম	বর্তমানে কর্মরত কিনা<br>1. হ্যাঁ<br>2.না	পেশা (পেশার কোড ব্যবহার করুন)	আয়<br>যেকোন একটি উত্তর হতে পারে<br>a) দিন এর গড় আয়, এবং মাসে কত দিন কাজ করে গড়ে মাসিক ইনকাম<br>*If household all work together in a family business and don't know individual income, put all income in household head's line. Tick if represents whole household income*	________ টাকা প্রতিদিন<br>(গত মাসে কত দিন কাজ করেছিলেন)___দিন<br><br>অথবা,<br><br>_____মাসিক আয়	________ টাকা প্রতিদিন<br>(গত মাসে কত দিন কাজ করেছিলেন)___দিন<br><br>অথবা<br><br>_____মাসিক আয়	Other source of Income & Amount in tk<br><br>1. Remittance (Once for a house)<br>2. Land<br>3. Pension<br>4. Rent<br>5. DPS Interest<br>6. Other
1							
2				☐			
3							
4	অর্থ প্রেরণ			________টাকা মাসিক			

121	পরবর্তী প্রশ্নটি কিভাবে আপনি পারিবারিক খরচ মিটিয়ে থাকেন সেই সম্পর্কে। কিভাবে আপনি পরিবারের প্রয়োজনীয় জিনিসপত্র কিনে থাকেন--প্রতিদিন<br>--প্রতি সপ্তাহ<br>--প্রতি মাস<br>--এবং বছরে এক/দুই বার<br><br>নির্দেশনা<br>যদি মেস এর কারো কাছ থেকে তথ্য নেয়া হয় সেই ক্ষেত্রে তার মেস এ কি খরচ হয় সেই হিসাব নিতে হবে (গ্রাম এর বাড়ির নয়)<br><br>গড়ে আপনার খানার মাসিক ব্যয় কত?<br>(যদি কোন খরচ না থাকে তাহলে ০০ লিখুন)<br><br>(পড়ে শোনান)	**(write daily/weekly/monthly as mentioned by respondent)**<br>1a.যাতায়াত -_________টাকা<br>1b.প্রতিদিনের খাবার(শাক–সবজি, মাছ, মাংশ)-_____ টাকা<br>2a.ঋণ/সঞ্চয় শোধ -_____ টাকা<br>3a.খাবার যেগুলো জমা করে রাখা যায় (চাল/ডাল)-_____ টাকা<br>3b.শিক্ষা খাতে খরচ (বেতন,কাগজ, কলম, বই, পোশাক)-_____ টাকা<br>3c.নিয়মিত চিকিৎসা খরচ -_____ টাকা<br>3d. বাসা ভাড়া বাবদ ------------<br>3e. গৃহের নিয়মিত খরচ (বিদ্যুৎ, পানি, জ্বালানি)-_____ টাকা<br>3f.মোবাইল খরচ -_____ টাকা<br>3g.আত্মীয়র র কাছে নিয়মিত ভাবে টাকা পাঠানো -_____ টাকা<br>4a.পোশাক-পরিচ্ছদ (জুতা,জামা, কাপড়)-_____ টাকা<br>4b.আসবাবপত্র (মোবাইল ফোন, ফ্রিজ, টেলিভিশন, ইত্যাদি)-_____ টাকা<br>4c.বড় ধরনের চিকিৎসা খরচ-_____ টাকা<br>4d.বিবিধ -_____ টাকা	

Start Time: _________	End Time: _________
Individual Respondent Module	
এই মডিউলটি সকল উপযুক্ত তথ্য প্রদান কারিদের উপর পরিচালনা করুন।<br><br>নিচের খালি জায়গায় উত্তরদাতার নাম ও লাইন নাম্বার পূরন করুন। আপনার নাম, নাম্বার, এবং তারিখ পূরন করুন।	
Case Line Number:<br>Case Age:<br>Respondent Line Number:<br>(if not the same person as the case)<br><br>Relationship to the case:	Intreviewer Name: ....<br><br>Code: ....<br>Date of interview: ....../............../2013
Result of the individual interview<br>Completed...1<br>Not at home .... .2<br>Refused... .3<br>Partly completed ....4<br>Disabled..5	

Sl	Questions	Code		Skip To
201	আপনি কতদিন যাবত এই শহরে বাস করছেন	A. নগরে কত মাস যাবত আছেন<br>__________<br>জানিনা 77	B. এই Colony/ moholla/para কত মাস যাবত আছেন _______<br>জানিনা 77	
202	আপনার গ্রামের বাড়ি কোথায়? [বিঃদ্রঃ জেলার নাম লিখুন]	_________________		
203	বর্তমান বৈবাহিক অবস্থা<br>(যদি বিবাহিত হয় তবে পুনরায় বিয়ে করেছে কিনা নিশ্চিত করুন)	1. বিবাহিত<br>2. আলাদা বসবাস<br>3. পরিত্যাক্ত<br>4. তালাকপ্রাপ্ত<br>5. বিধবা/বিপত্নীক<br>6. অবিবাহিত<br>7. পুনরায় বিবাহিত		
204	আপনি কি কখন ধর্মভিত্তিক শিক্ষা গ্রহন করেছেন?	1. হ্যাঁ<br>2. না		
204a	আপনি কি কোন সময় আনুষ্ঠানিক (স্কুল/মাদ্রাসা) শিক্ষা গ্রহন করেছেন?	1. হ্যাঁ<br>2. না		206
205	আপনি সর্বোচ্চ কোন ক্লাস পাস করেছেন??	ক্লাস..		
206	আপনি কতটা নিয়মিত খবরের কাগজ বা ম্যাগাজিন পড়েন?	1. প্রতিদিন<br>2. সপ্তাহে অন্তত একবার<br>3. অনিয়মিতভাবে<br>4. কখনই না		
207	আপনি কতটা নিয়মিত রেডিও শুনেন?	1. প্রতিদিন<br>2. সপ্তাহে অন্তত একবার<br>3. অনিয়মিতভাবে<br>4. কখনই না		209
208	আপনি সাধারনত কোন সময় রেডিও শুনেন?	1. সকালে<br>2. দুপুরে<br>3. বিকালে/সন্ধ্যায়<br>4. রাতে		
209	আপনি কতটা নিয়মিত টেলিভিশন দেখেন?	1. প্রতিদিন		

Sl	Questions	Code	Skip To
		2. সপ্তাহে অন্তত একবার<br>3. অনিয়মিতভাবে<br>4. কখনই না	211
210	আপনি সাধারনত কখন টেলিভিশন দেখেন?	1. সকালে<br>2. দুপুরে<br>3. বিকালে/সন্ধ্যায়<br>4. রাতে	
211	আপনার ধর্ম কি / আপনি কোন ধর্মের?	1. ইসলাম<br>2. হিন্দু / সনাতন ধর্ম<br>3. বৌদ্ধ<br>4. খ্রিস্টান<br>98. অন্যান্য (নির্দিষ্ট করে উল্লেখ করুন)---------	
211a	আপনি কি কোন ক্ষুদ্র নৃতাত্তিক জনগোষ্ঠীর অন্তর্ভুক্ত??	1. হ্যাঁ<br>2. না	
212	আপনার পেশা ...	1. প্রধান পেশা:<br>2.আনুষঙ্গিক/দ্বিতীয় পেশা:<br>(নিচে পেশার কোড দেখুন)	
213	গত চার সপ্তাহে আপনি কি নগদ টাকার বিনিময়ে কোন কাজ করেছেন?<br>যদি ২১২ নং প্রশ্নে বেকার অথবা গৃহিনী উলেস্নখ করা হয়।	1. হ্যাঁ<br>2. না	216
214	যদি হ্যাঁ হয় তবে কাজটি কি বাসার ভিতরে না বাহিরে ছিল?	1. ভিতরে / অভ্যন্তরে<br>2. বাহিরে	
215	আপনি কি ধরনের কাজ করেছিলেন?	________________<br>(নিচে পেশার কোড দেখুন)	
216	আপনার বাসা থেকে ১ কিঃমিঃ বা ১০-১৫ মিনিট পায়ে হাটা দুরত্বের মধ্যে কি কোন স্বাস্থ্য সেবা কেন্দ্র আছে?	1. হ্যাঁ<br>2. না<br>77.জানিনা	না অথবা জানিনা হলে 219 যান
217	যদি থাকে সেটি কোন ধরনের সেবাদানকারী প্রতিষ্ঠান / স্বাস্থ্য সেবা কেন্দ্র?<br>1. সরকারী হাসপাতাল/ ক্লিনিক<br>2. এনজিও<br>3. প্রাইভেট হাসপাতাল/ ক্লিনিক<br>4. ফার্মেসী<br>5. ডাক্তারের চেম্বার<br>6. ট্রেডিসনাল/ আধ্যাত্মিক চিকিৎসক<br>77.জানি না	নাম লিখুন এবং কোড গুলো নির্বাচন করুন<br>A.___________________1 2 3 4 5 6 77<br>B..___________________1 2 3 4 5 6 77<br>C. ___________________1 2 3 4 5 6 77<br>D. ___________________1 2 3 4 5 6 77<br>যদি আপনি না জানেন, তখন জানিনা উত্তরটি প্রযোজ্য।	
218	আপনি কিভাবে এই স্বাস্থ্য সেবাদানকারী প্রতিষ্ঠান বা স্বাস্থ্য কেন্দ্র সম্পর্কে জানতে পেরেছেন?<br>(একাধিক উত্তর গ্রহনয্যেগ্য)	1. পরিবার / বন্ধুবান্ধব<br>2. প্রতিবেশী<br>3.স্বাস্থ্যকর্মী<br>4. ফার্মাসিস্ট<br>5. ছাপান গণমাধ্যম<br>6. ইলেক্ট্রনিক গনমাধ্যম (টিভি, রেডিও)<br>7. সাইনবোর্ড<br>8.নিজ অভিজ্ঞতা<br>98. অন্যান্য (নির্দিষ্ট করে উল্লেখ করুন)	

Start Time: _________ End Time: _________

Core Health Care Seeking Behavior Module, Chronic Diseases

Enter eligible case's line number form HH listing:_____

আমি এখন আপনার কাছে কিছু স্বাস্থ্য সমস্যা স্পর্কে জানতে চাই যেগুলোতে দির্ঘদিন ধরে অথবা ঘন ঘন আক্রান্ত হচ্ছেন

Sl	Questions	Code		Skip To
1_300	Respondent line number			
2_300	Relationship to the case	1. Head-1<br>2. Spouse-2<br>3. Son /Daughter-3<br>4. Son-in-law/ Daughter-in-law-4<br>5. Grandchild-5<br>6. Parent-6<br>7. Parent-in-law-7<br>8. Sibling-in-law-8<br>9. Other relative-9<br>10. Adopted/ foster/ stepchild-10<br>11. Not related-11<br>12. Housekeeper-12<br>13. Brother/Sister<br>14. Self-<br>77. DK		
301	বর্তমানে আপনি/ আপনার বাচ্চা কি তিন মাসের অধিক কোন উপসর্গ লক্ষ্য করেছেন / স্বাস্থ্য সমস্যায় ভুগছেন? (probe for health problems)	1. হ্যাঁ<br>2. না		If yes at any, continue. Otherwise, end module.
302	আপনি/ আপনার বাচ্চা কি ঘন ঘন কোনো স্বাস্থ্য সমস্যায় ভুগছেন? (probe for health problems such as migraine)	1. হ্যাঁ<br>2. না		
303	আপনি/ আপনার বাচ্চা কি কোনো অক্ষমতায় ভুগছেন জার জন্য আপনাকে চিকিৎসা সেবা গ্রহন করতে হয়? (probe for disabilities)	1. হ্যাঁ<br>2. না		
304	301-302 উল্লেখিত সমস্যা গুলোর একটি ও কি MBBS ডাক্তার দ্বারা নির্নয় হয়েছে?<br>(নোট: অবশ্যই পাশ করা ডাক্তার হতে হবে, পল্লি চিকিৎসক বা ঔষধ বিক্রেতা দ্বারা নয়)	1. হ্যাঁ<br>2. না		
305	যদি হ্যাঁ হয় রোগ গুলো কি ছিল?<br>(রেকর্ড ধারন করুন)	___________<br>___________		
306	গত ৩ মাসে কি কি ধরনের উপসর্গ / স্বাস্থ্য সমস্যায় ভুগেছেন? (রেকর্ড ধারন করুন, পুঙ্খানুপুঙ্খভাবে তালিকাভুক্ত করুন)<br>কতদিন যাবৎ আপনি এই স্বাস্থ্য সমস্যায় ভুগছেন?	1.______________<br>2.______________<br>3.______________<br>4.______________<br>5._____________<br>6. ______________	1.__________ (d/w/m)<br>2.__________ (d/w/m)<br>3.__________ (d/w/m)<br>4.__________ (d/w/m)<br>5.__________ (d/w/m)<br>6.__________ (d/w/m)	
307	এর ভেতর কোনটি সবচেয়ে গুরুতর সমস্যা ছিল? (রেকর্ড / ধারন করুন)<br>**নিচের অসুস্থতা / উপসর্গের কোড গুলো ব্যাবহার করুন	_____________(___________)<br>কোড		
308	আপনার মতে এই সমস্যার কারন কি ?<br>(একাধিক উত্তর গ্রহনযোগ্য)<br>(প্রয়োজনে সবচেয়ে গুরুতর স্বাস্থ্য সমস্যার কথা মনে করিয়ে দিন)	1.বয়স<br>2. বাসায় কাজ করা<br>3. পেশা<br>4. দুর্ঘটনা<br>5. সংক্রামক<br>6. বংশগত<br>7. আশেপাসের পরিবেশ<br>77. জানি না<br>98. অন্যান্য (নির্দিষ্ট করে উল্লেখ করুন)_____		

Sl	Questions	Code	Skip To
309	এই সাস্থ্য সমস্যার জন্য আপনি কি করতে পারেন<br>(পড়ে শোনান)<br>কোডঃ<br>১= সহজেই / ১৬ আনা<br>২= সামান্য সমস্যা হয় / ১২ আনা,<br>৩= খুব সমস্যা হয় / ৮ আনা,<br>৪= কোন কাজই করেত পারিনা / মোটেও না / ০ আনা	A. নিজে নিজে খাওয়া......................... ১ ২ ৩ ৪<br>B. ভারী ওজন উঠানো………………..১ ২ ৩ ৪<br>C. ১ কিঃমিঃ হাটা…………...………১ ২ ৩ ৪<br>D. নোয়া, উবু হওয়া, হাটু গেড়ে বসা…..১ ২ ৩ ৪<br>E. কারো সাহায্য ছাড়া কাপড় পড়া …….১ ২ ৩ ৪<br>F. সাহায্য ছাড়া বাথরুমে যাও...........…১ ২ ৩ ৪<br>G. সাহায্য ছাড়াই বসা অবস্থান থেকে দাঁড়ানো……………………….….....…১ ২ ৩ ৪<br>H. সাহায্য ছাড়াই মাটিতে বসা অবস্থা থেকে দাঁড়ান ……………………………..……...…১ ২ ৩ ৪<br>I. ঘরের টুকিটাকি কাজ কর/স্কুলে / কাজে যাওয়া ......…………………………………..…১ ২ ৩ ৪	
310	আপনার এই স্বাস্থ্য সমস্যার জন্য আপনি কি কোন সেবা, পরামর্শ বা চিকিৎসা নিয়েছিলেন?<br>(বিঃদ্রঃ নিজ চিকিৎসা করে থাকলে হ্যাঁ হিসেবে বিবেচিত হবে)	1. হ্যাঁ<br>2. না	313
311	আপনি কেন এই অসুস্থতা / অক্ষমতার জন্য কোন সেবা গ্রহন করেননি?<br><br>(একাধিক উত্তর গ্রহনয্যেগ্য)	1.ভেবেছি নিজে নিজে ভাল হয়ে যাবে<br>2.গুরুতর ছিলনা<br>3. নিরাময়যোগ্য নয়<br>4. যাতায়াত খরচ বেশী<br>5. অনেক দূরে, কোন যানবাহন নেই<br>6. সেবাকেন্দ্রের দুর্বল নিরাপত্তা ব্যাবস্থা<br>7. নিরাপত্তাহীন ভ্রমন / যাতায়াত<br>8. সেবাসমূহ অতিরিক্ত ব্যায়বহুল<br>9. সেবাকেন্দ্রে কোন মহিলা সেবাপ্রদানকারী নেই<br>10. কর্মচারীদের অবন্ধুসুলভ আচরণ<br>11. সেবা প্রদানের সময় সুবিধাজনক নয়<br>12. ধর্মীয়/সামাজিক বিশ্বাস<br>13. সাথে যাওয়ার মতো কেউ নেই<br>14. কোথায় যেতে হবে জানতেন না<br>15. টাকা নেই<br>98.অন্যান্য (নির্দিষ্ট করুন)--------	
312	আপনি অসুস্থ্য হলে চিকিৎসা গ্রহনের/না গ্রহনের সিদ্ধান্ত কে নেয়?<br>(একাধিক উত্তর গ্রহনয্যেগ্য)	1.নিজে<br>2.স্বামী / স্ত্রী<br>3.বাবা / মা<br>4.শাশুড়ী<br>5.শ্বশুর<br>6.পরিবারের অনান্য সদস্য<br>98.অন্যান্য (নির্দিষ্ট করুন)---------	যদি ৩১১ এর উত্তর না হয় মডিউল সেশ করুন। And go to mobile use section
313	প্রশ্ন ৩০৬ এ তালিকাবদ্ধ সমস্যাগুলর মধ্যে কোনwU (কোনগু‡লার) জন্য আপনি শেষবার পরামর্শ বা সেবা নিয়েছিলেন?<br>(রেকর্ড করুন)		
314	শেষবার আপনার এই অসুস্থতা বা স্বাস্থ্য সমস্যার জন্য আপনি কোথায় কাছে গিয়েছিলেন?<br>(একাধিক উত্তর হতে পারে)	1.নিজেই চিকিৎসা করেছিলেন<br>2.প্রতিবেশী<br>3. বাসায় সেবা গ্রহন<br>4. ফামের্সী<br>5. সরকারী হাসপাতাল<br>6. প্রাইভেট ক্লিনিক/ হাসপাতাল<br>7. ডাক্তারের চেম্বার<br>8. এনজিও ক্লিনিক<br>98. অন্যান্য (নির্দিষ্ট করে লিখুন)	

Sl	Questions	Code	Skip To
314a	শেষবার আপনার এই অসুস্থতা বা স্বাস্থ্য সমস্যার জন্য আপনি কোথায় কাছে গিয়েছিলেন?<br>(most important one)	1.নিজেই চিকিৎসা করেছিলেন<br>2.প্রতিবেশী<br>3. বাসায় সেবা গ্রহন<br>4. ফার্মেসী<br>5. সরকারী হাসপাতাল<br>6. প্রাইভেট ক্লিনিক/ হাসপাতাল<br>7. ডাক্তারের চেম্বার<br>8. এনজিও ক্লিনিক<br>98. অন্যান্য (নির্দিষ্ট করে লিখুন)	
315	শেষবার আপনার এই অসুস্থতা বা স্বাস্থ্য সমস্যার জন্য আপনি কার কাছে গিয়েছিলেন?<br>(একাধিক উত্তর হতে পারে)	1.নিজেই<br>2. প্রতিবেশী (যিনি কোন স্বাস্থ্য সেবা প্রদানকারী নন)<br>3. ফার্মাসিস্ট/গ্রাম ডাক্তার<br>4. সনাতন চিকিৎসা(কবিরাজ, ফকির, ইমাম, ওঝা, হুযুর)<br>5.হোমিওপ্যাথিক ডাক্তার<br>6. MBBS ডাক্তার<br>7.স্বাস্থ্য কর্মী<br>8.মোবাইলের মাধ্যমে চিকিৎসা<br>a. ট্রেডিশনাল ডাক্তার<br>b. হোমিও ডাক্তার<br>c. এ্যালোপ্যাথ ডাক্তার<br>98. অন্যান্য (নির্দিষ্ট করে উল্লেখ করুন)____	
315a	শেষবার আপনার এই অসুস্থতা বা স্বাস্থ্য সমস্যার জন্য আপনি কার কাছে গিয়েছিলেন?<br>(most important one)	1.নিজেই<br>2. প্রতিবেশী (যিনি কোন স্বাস্থ্য সেবা প্রদানকারী নন)<br>3. ফার্মাসিস্ট/গ্রাম ডাক্তার<br>4. সনাতন চিকিৎসা(কবিরাজ, ফকির, ইমাম, ওঝা, হুযুর)<br>5.হোমিওপ্যাথিক ডাক্তার<br>6. MBBS ডাক্তার<br>7.স্বাস্থ্য কর্মী<br>8.মোবাইলের মাধ্যমে চিকিৎসা<br>a. ট্রেডিশনাল ডাক্তার<br>b. হোমিও ডাক্তার<br>c. এ্যালোপ্যাথ ডাক্তার<br>98. অন্যান্য (নির্দিষ্ট করে উল্লেখ করুন)____	
316	আপনি কিভাবে জানতে পেরেছিলেন যে এই অসুস্থতার জন্য আপনাকে কোথায় যেতে হবে / কি করতে হবে?*<br>(একাধিক উত্তর গ্রহনযোগ্য)	1.নিজের অভিজ্ঞ্যতা থেকে<br>2.স্বামী / স্ত্রী<br>3. পরিবারের অন্য সদস্য থেকে<br>4. বন্ধু/সহকর্মি<br>5. প্রতিবেশী<br>6. অন্য পরিচিত<br>7. নিকটস্থ ফার্মেসী থেকে<br>8. অন্য ডাক্তার এর মাধ্যমে<br>9. কোন মোবাইল স্বাস্থ্য লাইনের সাথে পরামর্শ করে<br>10. স্বাস্থ্যকর্মী<br>98. অন্যান্য (উল্লেখ করুন)________	
316a	আপনি কিভাবে জানতে পেরেছিলেন যে এই অসুস্থতার জন্য আপনাকে কোথায় যেতে হবে / কি করতে হবে?*<br>(most important one)	1.নিজের অভিজ্ঞ্যতা থেকে<br>2.স্বামী / স্ত্রী<br>3. পরিবারের অন্য সদস্য থেকে<br>4. বন্ধু/সহকর্মি<br>5. প্রতিবেশী<br>6. অন্য পরিচিত<br>7. নিকটস্থ ফার্মেসী থেকে<br>8. অন্য ডাক্তার এর মাধ্যমে<br>9. কোন মোবাইল স্বাস্থ্য লাইনের সাথে পরামর্শ করে<br>10. স্বাস্থ্যকর্মী	

Sl	Questions	Code	Skip To
		98. অন্যান্য (উল্লেখ করুন)________	
317	আপনি কেন এই সেবা প্রদানকারীকে / সেবাকেন্দ্রকে বেছে নিয়েছিলেন? *<br><br>(একাধিক উত্তর গ্রহনযোগ্য)	1. বাসার কাছে<br>2. স্বাস্থ্য সেবা প্রদানকারীর বন্ধুসুলভ আচরণ<br>3. পরিস্কার পরিচ্ছন্ন<br>4. সাশ্রয়ী/ কম খরচ/ফ্রি চিকিৎসা<br>5.টাকা নেই<br>6. ঔষধ সহজেই পাওয়া যায়<br>7. অন্যের পরামর্শের জন্য<br>8. ব্যাক্তিগত গোপনিয়তা রক্ষা করা হয়<br>9. মহিলা ডাক্তার উপস্থিতি<br>10. পাশ করা/বিশেষজ্ঞ ডাক্তার<br>11. বিশেষ দক্ষতা (যেমন হোমিওপ্যাথিক ডাক্তার)<br>12.কার্যকর চিকিৎসা<br>13. পূর্বের অভিজ্ঞতা থেকে<br>14. পরে পরিশোধ করতে পারবো (চিকিৎসা বা সেবার জন্য)<br>98. অন্যান্য (নির্দিষ্ট করে উল্লেখ করুন)___	
317a	আপনি কেন এই সেবা প্রদানকারীকে / সেবাকেন্দ্রকে বেছে নিয়েছিলেন? *<br><br>(most important one)	1. বাসার কাছে<br>2. স্বাস্থ্য সেবা প্রদানকারীর বন্ধুসুলভ আচরণ<br>3. পরিস্কার পরিচ্ছন্ন<br>4. সাশ্রয়ী/ কম খরচ/ফ্রি চিকিৎসা<br>5.টাকা নেই<br>6. ঔষধ সহজেই পাওয়া যায়<br>7. অন্যের পরামর্শের জন্য<br>8. ব্যাক্তিগত গোপনিয়তা রক্ষা করা হয়<br>9. মহিলা ডাক্তার উপস্থিতি<br>10. পাশ করা/বিশেষজ্ঞ ডাক্তার<br>11. বিশেষ দক্ষতা (যেমন হোমিওপ্যাথিক ডাক্তার)<br>12.কার্যকর চিকিৎসা<br>13. পূর্বের অভিজ্ঞতা থেকে<br>14. পরে পরিশোধ করতে পারবো (চিকিৎসা বা সেবার জন্য)<br>98. অন্যান্য (নির্দিষ্ট করে উল্লেখ করুন)___	
318	আপনি এই অসুস্থতার জন্য কতবার স্বাস্থ্যসেবা প্রদানকারীকে দেখিয়েছেন?<br>(skip if self treatment: Q 316 option 1)	1.প্রতি মাসে বা বেশী<br>2. প্রতি ২ -3 মাস<br>3. প্রায়ই 4-5 মাস<br>4. বছরে এক বা দুইবার<br>5. বছরে একবারের কম<br>98. অন্যান্য (উল্লেখ করুন)________	
319	শেষবার চিকিৎসা নেয়ার সময় আপনাকে কত টাকা খরচ করতে হয়েছিল?<br>(skip if self treatment: Q 316 option 1)<br><br>(প্রত্যেক খরচ সম্বন্ধে আলাদা আলাদা করে জিজ্ঞেস করম্নন, যদি উত্তরদাতা আলাদা আলাদা কওে উত্তর দিতে অক্ষম হয়, তাহলে মোট খরচ কত হয়েছিল তা জিজ্ঞেস করম্নন।)	1. যাতায়াত খরচ-<br>2. রেজিস্ট্রেশন ফি-<br>3. ডাক্তারের ফি/হসপাতাল খরচ-<br>4. ল্যাবের পরীক্ষা সমূহ-<br>5. মেডিসিন খরচ সরবরাহ -<br>6. অলিখিত খরচ (ঘুষ)<br>7. অলিখিত খরচ (বকশিস)<br>8. প্যাকেজ খরচ-<br>9. অন্যান্য-<br>10. মোট খরচ-<br>11. কোন টাকা লাগেনি<br>77. জানি না	
320	আপনি কিভাবে এই চিকিৎস্যা খরচ মিটিয়েছিলেন?	1.পারিবারিক আয় থেকে<br>2.পারিবারিক সঞ্চয় থেকে<br>3.সম্পদ বিক্রি করে<br>4.ঋণ গ্রহন করে (ন জি ও)<br>5.ঋণ গ্রহন করে (স্থানীয় মহাজন থেকে)	

Sl	Questions	Code	Skip To
		6.অনুদান গ্রহন করে (প্রতিবেশী/বন্ধু/আত্মীও)<br>7.অনুদান গ্রহন করে (ন জি ও)<br>৮. ঋণ গ্রহন (প্রতিবেশী, আত্মীয়, বন্ধু)<br>98. অন্যান্য(উল্লেখ করুন)	
321	আপনি চিকিৎস্যা বাবদ মাসে কত টাকা খরচ করে থাকেন? (ঔষধ ,ডাঃ ফি, যাতায়াত, ইত্যাদি সহ)	----------টাকা	
