# Supplementary material for: Healthcare seeking for chronic illness among adult slum dwellers in Bangladesh: A descriptive cross-sectional study in two urban settings
Source: PLoS One. 2020 Jun 15;15(6):e0233635. doi: 10.1371/journal.pone.0233635 (PMC7295220; doi:10.1371/journal.pone.0233635)
Supplement: S2 File — (PDF) [file pone.0233635.s002.pdf]

Indicate the result of the household survey in the household listing provided
"*" Signifies multiple response for a question.

**HOUSEHOLD IDENTIFICATION**	
City Corporation:………………………………………………………………….<br>Cluster Number..<br>Household Number..<br>Type of Household: 1. Family 2. Mess<br>Name of The Household Head………………………………………..<br>Name of The Respondent………………………………………	Household GPS Location:<br>___ ____ ____ ___.____ ____

**INTERVIEW DETAILS**	
Interviewer Code:<br>Result<br>1st Visits: ___/____/______ Start Time: ___:____ End Time:_____:____<br>2nd Visits:____/______/____ Start Time: ___:____ End Time:_____:____<br>3rd Visits:____/______/____ Start Time: ___:____ End Time:_____:____	Interview Completed<br>1. Yes<br>2. No

Cross Checker:	Date of Cross Check: ___/______/___

Supervisor: ____________________<br>Date: _____/______/________	Field Editor:__________________<br>Date: _____/______/________	Remarks

Healthcare Seeking Behavior Among the Urban Poor in Bangladesh, 2013
Screening Questionnaire for Household Membership Listing

*Eligibility Criteria (Code):
1= Individual Respondent Module (For All)
2= Health Care Seeking Behavior Module (15 yr and Above)
3= Not Eligible

SL	101	102	103	104	105	106	106a	107	107a	107b	107b_1	107c	107c_1	107d	107e	107f	107g	107h	107i	107j
	Name	Sex	Age	Marital Status	Relation to the Household Head**	Mother who has delivered baby within last 1 Year	Are you currently pregnant	Mother/Guardian who has living child age under 5 years	Mother/Guardian who has living child age between 5-14 years	Health problem for last 14 days	Specify the Disease	Health problem for last 3 months or more	Specify the Disease	Any physical/ mental disability	If yes, have you sought treatment in last 3 months?	Unable speak or hear?	Eligibility for Module *	Available in HH for next 2 weeks?	Available Appointment Time	Eligible for module*
		1=M<br>2=F	-----/-----<br>y/m	1. Married<br>2.Separated<br>3. Divorced<br>4.Widowed<br>5.Unmarried	*See the options below the table***	1=Yes<br>2=No<br>3= NA	1=Yes<br>2=No	1=Yes<br>2=No<br>3= NA	1=Yes<br>2=No<br>3= NA	1=Yes<br>2=No<br>3= NA		1=Yes<br>2=No<br>3= NA		1=Yes<br>2=No	1=Yes<br>2=No	1=Yes<br>2=No	*See the Codes above **			
1.																				
2.																				
3.																				
4.																				
5.																				
6.																				
7.																				
8.																				
9.																				
10.																				

**01 = Head, 02 = Spouse, 03 = Son /Daughter, 04 = Son-in-law/ Daughter-in-law, 05 = Grandchild, 06 = Parent, 07 = Parent-in-law, 08 = Sibling-in-law, 09 = Other relative, 10 =Adopted/ foster/ stepchild, 11 = not related12=housekeeper, 13= Brother/Sister 77 = DK

	**SOCIOECONOMICCHARACTERSTICS**<br>DO NOT READ THE OPTION OF ANSWER UNLESS THERE IS A SPECIFIC INSTRUCTION			
	Indicator	Value	Points	Score
108	How many household members are 12-years-old or younger?	A. Three or more<br>B. Two<br>C. One<br>D. None	0<br>10<br>16<br>29	
109	Do all household members aged 6-to-12 currently attend a school/educational institution?	A. No<br>B. No one 6-to-12<br>C. Yes	0<br>3<br>6	
110	In the past year, did any household member ever do work for which he/she was paid on a daily basis?	A. Yes<br>B. No	0<br>8	
111	How many rooms does your household occupy (excluding rooms used for business)?	A. One<br>B. Two<br>C. Three or more	0<br>3<br>5	
112	What is the main construction material of the walls of the main room?	A. hay/bamboo, or other<br>B. Tin/wood<br>C. Brick/cement/ Concrete	0<br><br>2<br><br>9	
113	Does the household own any televisions?	A. No<br>B. Yes	0<br>7	
114	How many fans does the household own?	A. None<br>B. One<br>C. Two or more	0<br>4<br>7	
115	How many mobile phones does the household own?	A. None<br>B. One<br>C. Two or more	0<br>8<br>15	
116	Does the household own any bicycles, motorcycle/scooters, or motor cars etc.?	A. No<br>B. Yes	0<br>4	
117	Does the household own (or rent/sharecrop/mortgage in or out) 51 or more decimals of cultivable agricultural land (excluding uncultivable land and dwelling-house/homestead land)?	A. No<br>B. Yes	0<br>7	

Note: Using the Total Score find PPI from PPI scorecard.

TICK HERE IF CONTINUATION SHEET USED	
Just to make sure that I have a complete listing:	
Are there any other persons such as small children or infants that we have not listed?<br>(If YES Go back to household schedule and enter new members in the household schedule.)	
In addition, are there any other people who may not be members of your family, such as domestic servants, lodgers or friends who usually live here?<br>(If YES Go back to household schedule and enter new members in the household schedule.)	

Sl	Questions	Code			Skip To
119	Does your household have: (read out)<br>(at the current residence)		YES	NO	
		ELECTRICITY	1	2	
		WORKING RADIO	1	2	
		WORKING TELEVISION	1	2	
		MOBILE TELEPHONE	1	2	
		REFRIGERATOR	1	2	
		BICYCLE	1	2	
		MOTORCYCLE	1	2	
		CAR OR TRUCK	1	2	
		RICKSHAW/VAN	1	2	
		DVD PLAYER	1	2	

Household Member Occupation and Income<br>*If mess- only record individual's income*							
LINE NO.	101<br>Select Member (name)	120b<br>Are they working?<br>What is their occupation?<br>(Refer to occupation code)<br>Don't assume- prompt for all	120c<br>Represents whole household<br>1=Yes<br>2=No<br>*If household all work together in a family business and don't know individual income, put all income in household head's line.*	120d<br>Income Type<br>1.Daily<br>2. Monthly	120f<br>How many days did you work last month	120g<br>Do you have Other source of Income<br>1=Yes<br>2=No	120h<br>Other source of Income & Amount in tk<br>1. Remittance (Once for a house)<br>2. Land<br>3. Pension<br>4. Rent<br>5. DPS Interest<br>6. Other
1.							
2.							
3.							
4.							
5.							
6.							
7.							
8.							
9.							
10.							

121	This next question asks about the different things that you have to pay for in your household. I'm interested in the things that you buy<br>… every day<br>… every week<br>… every month<br>… and about once a year<br><br>Tab has been set to allow the interview to enter the time period volunteered by the respondent, then the taka spent in that time period.<br><br>Instruction: If living in a mess – ensure they are answering for their OWN expenditure in the mess (and not in their village home)	**(write daily/weekly/monthly as mentioned by respondent)**<br>1.a Transport<br>1.b Daily food (vegetables, meat and fish)<br>2.a Loan repayments<br>3.a Food that can be stored (rice, dahl)<br>3.b Educational costs (tuition, stationary, books, uniforms)<br>3.c Regular medical expenses (medication)<br>3.d House rent-<br>3.e Housing and utilities (electricity, gas, water)<br>3.f Mobile phone bills<br>3.g Money that you send to relatives<br>4.a Clothing and footwear<br>4.b Furniture and electronics (mobile phone, television, etc)<br>4.c Large medical expenses<br>4.d Miscellaneous expenses (large one-off)	

**Start Time:** _________	**End Time:** _________

**Individual Respondent Module**	
This module is to be administered to all eligible respondents.<br><br>*Fill in the cluster and household number, and the name and line number of the individual in the space below. Fill in your name, number and the date.*	
1_200 Respondent Line Number:<br>2_200 Relationship to case:<br>3-200 Case Age:<br>4_200 Need for this module: 1- Yes, 2- No<br><br>Case Age:<br><br>[illegible]	Interviewer Name....................................<br>Code..........<br>Date of interview: 2013
Result of the individual interview<br>Completed...1<br>Not at home .... .2<br>Refused... .3<br>Partly completed ....4<br>Disabled...5<br>Other (*specify*) ……………………………………96	City Corporation:<br><br>Cluster Number:<br><br>Household Number:

NO.	QUESTIONS	CODE		SKIP
201	How long have you been living continuously in this -	**a. City Corporation**<br>Completed months___<br>DK--77	**b.** Colony/ moholla/para<br>Completed months___<br>DK--77	
202	Where is your home district?<br>Note: Write name	________________		
203	Current marital status<br><br>**(IF MARRIED PROBE FOR WHETHER REMARRIED OR NOT)**	1. Married<br>2. Separated<br>3. Deserted<br>4. Divorced<br>5. Widowed/Widower<br>6. Never married<br>7. Remarried		
204a	Have you ever attended religious education?	1. Yes<br>2. No		
204b	Have you ever attended formal education (school/madrasa)?	1. Yes<br>2. No		 206
205	What is the highest class you completed?	(Completed highest class) ……………….		
206	How often do you read newspaper or magazine?	1. Every day<br>2. At least once a week<br>3. Infrequently<br>4. Never		
207	How often do you listen to the radio?	1. Every day<br>2. At least once a week<br>3. Infrequently<br>4. Never		 <br><br>209
208	When do you usually listen to radio?	1. Morning<br>2. Afternoon		

		3. Evening<br>4. Night	
209	How often do you watch television?	1. Every day<br>2. At least once a week<br>3. Infrequently<br>4. Never	 <br><br>211
210	When do you usually watch television?	1. Morning<br>2. Afternoon<br>3. Evening<br>4. Night	
211	What is your religion?	1. Islam<br>2. Hinduism<br>3. Buddhism<br>4. Christianity<br>98.Other(specify) _______________	
211a	Do you belong to any minority ethnic group?	1. Yes<br>2. No	
212	What is your….	a. principle occupation:<br>b. secondary occupation:<br><br>occupation code (see below)	
213	Have you done any work for cash in the last four weeks? Ask if mentioned unemployed or housewife in 212	1. Yes<br>2. No	 216
214	Was this work inside or outside the home?	1. Inside<br>2. Outside	
215	What kind of work did you do?	________________<br>occupation code (see below)	
216	Are there any health SERVICE PROVIDERS/FACILITIES available within 1 kilometer/10-15 minutes of walking distance from your home?	1. Yes<br>2. No<br>77. DK	219 for No OR DK
217	What health SERVICE PROVIDERS/FACILITIES are available within 1 kilometer/10-15 minutes of walking distance from your home?<br>CODE:<br>1. GOVT. Hospital/clinic<br>2. NGO<br>3. Private. Hospital/clinic<br>4. Pharmacy<br>5. Doctor's chamber<br>6. Traditional/spiritual healers<br>77. DK	Write Names & Select Code<br><br>A.___________________1 2 3 4 5 6 77<br><br>B..___________________1 2 3 4 5 6 77<br><br>C. ___________________1 2 3 4 5 6 77<br><br>D. ___________________1 2 3 4 5 6 77<br><br>(if don't know name, put "don't know")	

218	How do you know about this/these facility/facilities, providers? *	1. Family/friends<br>2. Neighbors<br>3. Health workers<br>4. Pharmacist<br>5. Print Media (News paper)<br>6. Electronic media (TV, Radio, etc.)<br>7. Signboard<br>8. Own experience<br>98. Other(specify) _______________	
		98.Other(specify) _______________	
218a	How do you know about this/these facility/facilities, providers? (Most important one)	1. Family/friends<br>2. Neighbors<br>3. Health workers<br>4. Pharmacist<br>5. Print Media (News paper)<br>6. Electronic media (TV, Radio, etc.)<br>7. Signboard<br>8. Own experience<br>98.Other(specify) _______________	

Core Health Care Seeking Behavior Module (CHRONIC DISEASES)				
**NO.**	**Now I would like to ask about any long-term or repeated health conditions you are experiencing**	**CODE**		**SKIP To**
1_300	Respondent line number			
2_300	Relationship to the case	1. Head-1<br>2. Spouse-2<br>3. Son /Daughter-3<br>4. Son-in-law/ Daughter-in-law-4<br>5. Grandchild-5<br>6. Parent-6<br>7. Parent-in-law-7<br>8. Sibling-in-law-8<br>9. Other relative-9<br>10. Adopted/ foster/ stepchild-10<br>11. Not related-11<br>12. Housekeeper-12<br>13. Brother/Sister<br>14. Self-<br>77. DK		
301	Are you/your child currently suffering from a long-term health problem that has lasted more than three months?<br><br>Probe for health problems	1. Yes<br>2. No		If yes at any, continue. Otherwise, end module.
302	Do you suffer from any health problem that comes and goes repeatedly (such as migraine)<br><br>Probe for health problems	1. Yes<br>2. No		
303	Do you suffer from any disabilities that require medical treatment?<br><br>Probe for disabilities	1. Yes<br>2. No		
304	Have you been diagnosed (by an MBBS doctor) for any of these problem?<br><br>(note- must be qualified- not pharmacist or village doctor)	1. Yes<br>2. No		
305	(If yes) what were the diagnoses?<br>(record verbatim)	________________<br>_______________<br>________________		
306	What are the symptoms/health problems you have been experiencing for last three or more months?<br><br>For each- how long have you had these symptoms? (answered in days/weeks/months)<br><br>(record and list verbatim)	1.________________<br>2.________________<br>3.________________<br>4.________________<br>5.________________<br>6.________________	1.__________ (d/w/m/y)<br>2.__________ (d/w/m/y)<br>3.__________ (d/w/m/y)<br>4.__________ (d/w/m/y)<br>5.__________ (d/w/m/y)<br>6.__________ (d/w/m/y)	
307	Which one is the most severe symptom/health problem?<br>(record)	_____________(___________)<br>Code		

308	In your opinion what could be the causes of this symptom?*<br><br>Note- remind referring to most severe symptom if necessary	1. Age<br>2. Work at home<br>3. Occupation<br>4. Accident<br>5. Contagious<br>6. Family history/genetic<br>7. Living environment<br>77. DK<br>98.Other specify_______________	
308_1	In your opinion what is the most important cause of this symptom?	1. Age<br>2. Work at home<br>3. Occupation<br>4. Accident<br>5. Contagious<br>6. Family history/genetic<br>7. Living environment<br>77. DK<br>98.Other specify_______________	
308a	Age category	1. More than 14 years<br>2. 0-14 years	
309	Due to this symptom are you able to **(READ OUT)**<br><br>Code:<br>1= Easily/16 ana,<br>2= With Slight Difficulty/12 ana,<br>3= With Much Difficulty/8 ana,<br>4= Not at all/0 ana<br><br>Note- remind referring to most severe symptom if necessary	A. Feed yourself................................1 2 3 4<br>B. Carry a heavy load..........……..…1 2 3 4<br>C. Walk 1 kilometer..........................1 2 3 4<br>D. Bow, squat, kneel.........................1 2 3 4<br>E. Dress without help........................1 2 3 4<br>F. Go to the bathroom without help....1 2 3 4<br>G. Stand up from a sitting position without help……………………………..….…1 2 3 4<br>H. Stand up from sitting on the floor without help……………………………..........1 2 3 4<br>I. Perform household chores/ Attend school/work ……………………………………....1 2 3 4	
310	Did you seek any care, advice or treatment for any of your symptoms?<br>(Note: Self care should be regarded as Yes. Refers to *any* of the symptoms mentioned at 306)	1. Yes<br>2. No	313<br>312
311	Why didn't you seek care for this health problem or disability?*<br>*(skip if did seek treatment)*	1. Thought would go away by itself<br>2. Not severe<br>3. Not curable<br>4. Transport too expensive<br>5. Too far, no transportation<br>6. Poor security at facility<br>7. Insecure travel<br>8. Services too expensive/cannot afford<br>9. No female provider at facility<br>10. Unfriendly staff<br>11. Inconvenient service hours<br>12. Religious or cultural belief<br>13. No one to accompany<br>14. Didn't know where to go<br>15, Lack of money<br>98.Others, specify_______________	

312	Who made the decision about seeking/not seeking treatment?*	1. Self<br>2. Spouse<br>3. Parent<br>4. Mother-in-law<br>5. Father-in-law<br>6. Other family members<br>98.Others, specify: _______________	If no at 311, finish chronic section.
312a	Who made the decision about seeking/not seeking treatment? (Most important one)	1. Self<br>2. Spouse<br>3. Parent<br>4. Mother-in-law<br>5. Father-in-law<br>6. Other family members<br>7. 98.Others, specify: _______________	
313	For which one (or more) symptoms/health problems mentioned in Q 306 did you seek advice/care the last time? (record)	___________________	
314	Where did you go for this health problem the last time?*	1. Self-treatment<br>2. Neighbour<br>3. Door step service<br>4. Pharmacy<br>5. Government hospital<br>6. Private clinic / hospital<br>7. Doctor's chamber<br>8. NGO clinic<br>98.Others, specify: _______________	
314a	Where did you go for this health problem the last time? (most important one)	1. Self-treatment<br>2. Neighbour<br>3. Door step service<br>4. Pharmacy<br>5. Government hospital<br>6. Private clinic / hospital<br>7. Doctor's chamber<br>8. NGO clinic<br>98.Others, specify: _______________	
315	Who did you go to for this health problem the last time?*	1. Self treatment<br>2. Neighbor (not a health professional)<br>3. Pharmacist /Village doctor<br>4. Traditional healer<br>5. Homeopath doctor<br>6. MBBS doctor<br>7. Community health worker (SK/CSBA)<br>8. Over mobile phone<br>a. Traditional healer<br>b. Homeopath doctor<br>c. Allopath doctor<br>98.Others, specify _______________	
315a	Who did you go to for this health problem the last time? (most important one)	1. Self treatment<br>2. Neighbor (not a health professional)<br>3. Pharmacist /Village doctor<br>4. Traditional healer<br>5. Homeopath doctor<br>6. MBBS doctor<br>7. Community health worker (SK/CSBA)<br>8. Over mobile phone<br>a. Traditional healer<br>b. Homeopath doctor<br>c. Allopath doctor<br>98.Others, specify _______________	

316	How did you know where to go/what to do for this problem(s)?*	1. From my own experience<br>2. Spouse<br>3. From other family members<br>4. From friends/From colleagues<br>5. From neighbours<br>6. From other acquaintances<br>7. From local pharmacy<br>8. From another doctor (referral)<br>9. Consultation with any mobile health line<br>10. From a community health worker (SK)<br>98. Others, specify:_______________	
316a	How did you know where to go/what to do for this problem(s)? (most important one)	1. From my own experience<br>2. Spouse<br>3. From other family members<br>4. From friends/From colleagues<br>5. From neighbours<br>6. From other acquaintances<br>7. From local pharmacy<br>8. From another doctor (referral)<br>9. Consultation with any mobile health line<br>10. From a community health worker (SK)<br>98. Others, specify:_______________	
317	Why did you choose this provider/facility?*	1. Close to home<br>2. Provider friendly<br>3. Clean<br>4. Low cost/ free<br>5. Lack of money<br>6. Medicines available<br>7. Recommended by others<br>8. Privacy maintained<br>9. Female doctors available<br>10. Qualified doctors (MBBS or specialist)<br>11. Special skills (such as homeopathic doctor)<br>12. Effective treatment<br>13. Prior experience<br>14. Can pay later (for medication, treatment)<br>98. Others, specify _______________	
317a	Why did you choose this provider/facility? (most important one)	1. Close to home<br>2. Provider friendly<br>3. Clean<br>4. Low cost/ free<br>5. Lack of money<br>6. Medicines available<br>7. Recommended by others<br>8. Privacy maintained<br>9. Female doctors available<br>10. Qualified doctors (MBBS or specialist)<br>11. Special skills (such as homeopathic doctor)<br>12. Effective treatment<br>13. Prior experience<br>14. Can pay later (for medication, treatment)<br>98. Others, specify _______________	
318	How often do you see a health provider for this health problem? (skip if self-treatment: Q316 option 1)	1. Every month or more frequently<br>2. Every 2-3 months<br>3. Every 4-5 months<br>4. 1-2 times a year<br>5. Less than once a year<br>98. Other, specify_______________	

319	How much did you have to spend for the last visit?<br>RECORD THE AMOUNT IN TAKA.<br><br>ASK EACH ITEM SEPARATELY. IF A RESPONDENT IS UNABLE TO REMEMBER SEPARATELY HOW MUCH HE PAID FOR EACH ITEM, THEN ASK FOR A TOTAL.	1. Transport costs---------------<br>2. Registration fees------------<br>3. Consultation/ hospitalization fees--<br>4. Lab tests-----------------------------<br>5. Medicine & supplies-------------<br>6. Unofficial payments (bribes)-------<br>7. Unofficial payments (tips)---<br>8. Package treatment(expenses together)<br>9. Other<br>10. Total<br>11. No expenses<br>77. DK	
320	How did you pay for this?*	1. From household wages<br>2. Savings<br>3. Selling household objects<br>4. Borrowing money from NGO<br>5. Borrowing money from money lender<br>6. Donation from a neighbor, friend or relative<br>7. Donation from NGO<br>8. Borrowing money form family/relative/friend<br>98. OTHERS(SPECIFY) _________________	
320	How did you pay for this? (most important on	1. From household wages<br>2. Savings<br>3. Selling household objects<br>4. Borrowing money from NGO<br>5. Borrowing money from money lender<br>6. Donation from a neighbor, friend or relative<br>7. Donation from NGO<br>8. Borrowing money form family/relative/friend<br>98.OTHERS(SPECIFY) _________________	
321	How much do you spend monthly for medicines, consultation, and supplies to address this health problem (including transport costs and all doctor's fees)?	_________ Taka	
